# Delineating Crosstalk Mechanisms of the Ubiquitin Proteasome System That Regulate Apoptosis

**DOI:** 10.3389/fcell.2018.00011

**Published:** 2018-02-09

**Authors:** Ishita Gupta, Kanika Singh, Nishant K. Varshney, Sameena Khan

**Affiliations:** ^1^Structural Immunology Group, International Centre for Genetic Engineering and Biotechnology, New Delhi, India; ^2^Drug Discovery Research Centre, Translational Health Science and Technology Institute, Faridabad, India

**Keywords:** apoptosis, DUBs, E3 ligases, ubiquitination, ubiquitin proteasome system

## Abstract

Regulatory functions of the ubiquitin-proteasome system (UPS) are exercised mainly by the ubiquitin ligases and deubiquitinating enzymes. Degradation of apoptotic proteins by UPS is central to the maintenance of cell health, and deregulation of this process is associated with several diseases including tumors, neurodegenerative disorders, diabetes, and inflammation. Therefore, it is the view that interrogating protein turnover in cells can offer a strategy for delineating disease-causing mechanistic perturbations and facilitate identification of drug targets. In this review, we are summarizing an overview to elucidate the updated knowledge on the molecular interplay between the apoptosis and UPS pathways. We have condensed around 100 enzymes of UPS machinery from the literature that ubiquitinates or deubiquitinates the apoptotic proteins and regulates the cell fate. We have also provided a detailed insight into how the UPS proteins are able to fine-tune the intrinsic, extrinsic, and p53-mediated apoptotic pathways to regulate cell survival or cell death. This review provides a comprehensive overview of the potential of UPS players as a drug target for cancer and other human disorders.

## Introduction

Protein ubiquitination is a regulatory posttranslational modification (PTM) assuming an important position in balancing homeostasis of the cell. Ubiquitination is an attachment of ubiquitin (~76 amino acid) tag to the substrate protein, which then presents a way to control the functions and stability of cellular proteins, their localization and sometimes the fate of the cell. Ubiquitination is the main signal in a cell that balances the myriad intracellular proteins levels and partakes in major cellular processes likes of apoptosis, DNA replication and repair, cell cycle progression, transcription, and immune responses, to name a few, by inducing the selective proteolysis via 26S proteasome (Argentini et al., 2000; Huang et al., 2000; Suzuki et al., 2001; Wilson et al., 2002; Haglund and Dikic, 2005; Nakayama and Nakayama, 2006; Mukhopadhyay and Riezman, 2007; Ikeda and Dikic, 2008). The ubiquitin-proteasome system (UPS) consists mainly of E3 ligases and deubiquitinating enzymes (DUBs) are the key regulator of the apoptosis process by regulating the pro or antiapoptotic proteins and dictate the cell survival vs. death. Briefly, apoptosis is a physiological cell death that is mediated by caspase family of intracellular cysteine proteases and is critically influential in the normal development and function of multicellular organisms. The pathway involves the assembly of signaling complexes that culminates into the cell death (Vucic et al., 2011). Modification of these complexes by the addition of ubiquitin tag often destabilizes them and target for proteasomal degradation. Nearly 700 E3 ligases (Li et al., 2008) and 100 DUBs (Nijman et al., 2005; Komander et al., 2009) are encoded by the human genome and crosstalk of those with apoptotic pathways has been observed which ultimately governs the cell fate. Given the astounding importance of UPS in regulating the wide array of cellular processes, deregulation of the UPS has been associated with the disruption of cellular homeostasis and eventually resulted in the genesis of a number of disorders and tumors (Nalepa and Harper, 2003; Ciechanover, 2006; Reinstein and Ciechanover, 2006; Paul, 2008; Popovic et al., 2014). After the success of proteasomal inhibitors in the treatment of multiple myeloma, components of the UPS are being researched as drug targets against other malignancies as well as metabolic and neurodegenerative diseases. As a result, novel UPS enzymes and their adaptors have been identified as conceivable targets for the development of more selective and specific therapeutics (Adams, 2002; Orlowski et al., 2002; Nalepa and Harper, 2003; Issaeva et al., 2004; Hoeller et al., 2006; Richardson et al., 2006; Deshaies, 2014; Huang and Dixit, 2016).

This review intends to delineate the two complex pathways: the ubiquitin-proteasome system and apoptosis. We have summarized around 100 UPS enzymes from the plethora of literature that ubiquitinates and/or deubiquitinates various proteins exclusively either of intrinsic, extrinsic, or p53-mediated apoptotic pathways and ultimately influences the cell fate. We have gathered and compiled the mechanistic details of E3s and DUBs affecting above-mentioned pathways on their substrates, however for many, little is known as the field is in its infancy. We have attempted to list those E3s and DUBs as well where their expression levels in the cells shown to influence the expression of many apoptotic factors, which eventually dictate the cell fate. There are many apoptotic proteins that are modulated by multiple E3 ligases and DUBs; some of them are expressed differentially either with respect to the different cell line or stimuli. Studies have proved that when one apoptotic factor is regulated by more than one E3s or DUBs, they either work in cooperation or in some case, stimulate the activity of other. Similarly, many E3s and DUBs usually have several substrates and are cell specific. It is far regarded that the UPS components i.e., E1s, E2s, E3s, DUBs, and proteasome are potential drug targets to treat several diseases. We have discussed some of the advancement in targeting UPS as therapeutics in various malignancies, metabolic, and neurodegenerative diseases.

## The UPS machinery

**Figure 1:**
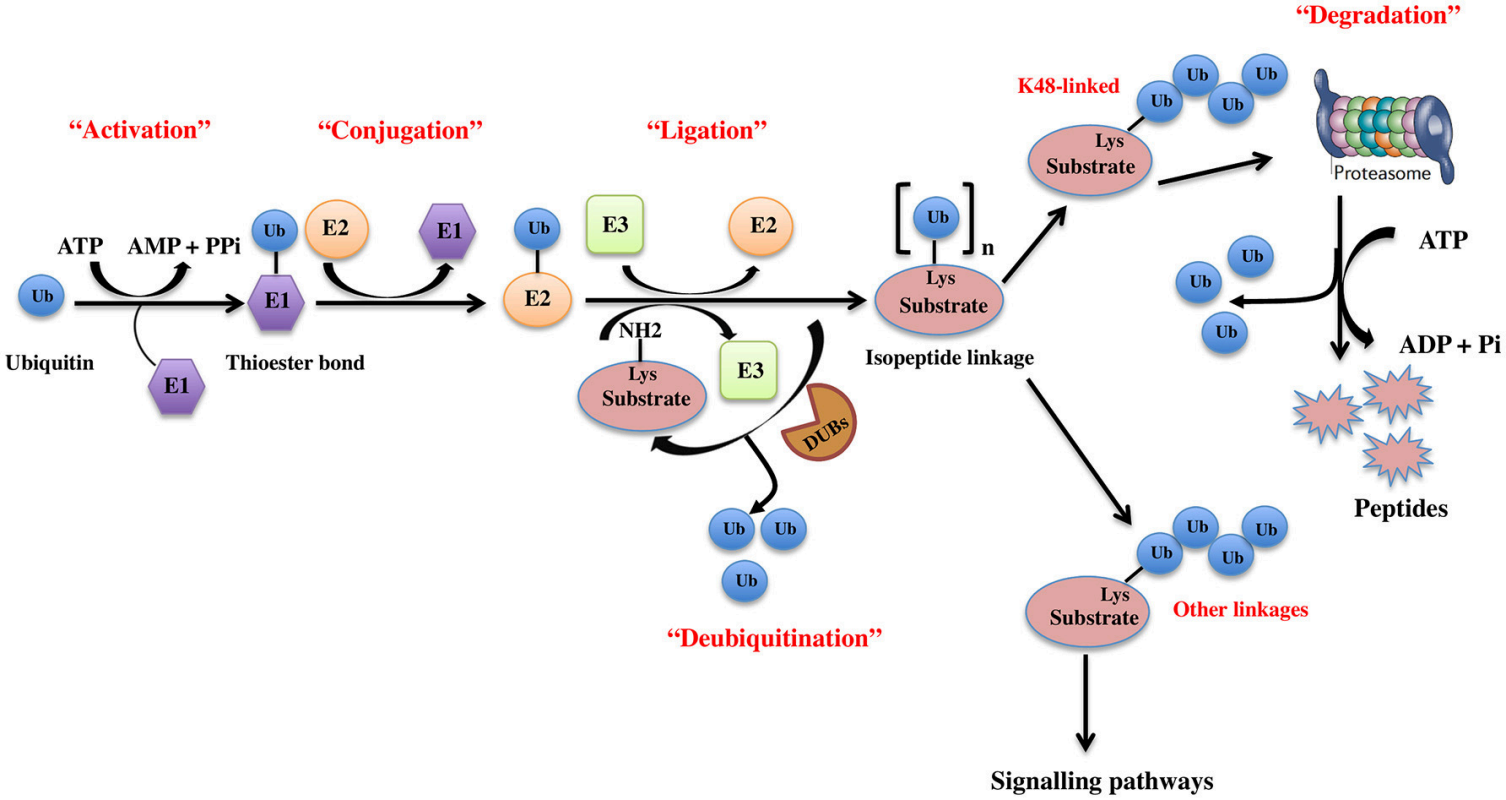
The ubiquitin pathway. Ubiquitination is the covalent addition of a 76-amino acid ubiquitin tag to the N-terminal lysine of the substrate protein. This process requires the sequential actions of three enzymes, an activating enzyme (E1) that forms an ATP-dependent labile thioester linkage with the carboxyl-terminal group of ubiquitin through its cysteine thiol group, thereby activating the C-terminus of ubiquitin for nucleophilic attack; a conjugating enzyme (E2) that transiently carries the activated ubiquitin molecule as a thiol ester; and an ubiquitin ligase (E3) that finally transfers the activated ubiquitin from the E2 to the ε-amino group of acceptor lysine residue of the substrate.

**Figure 2:**
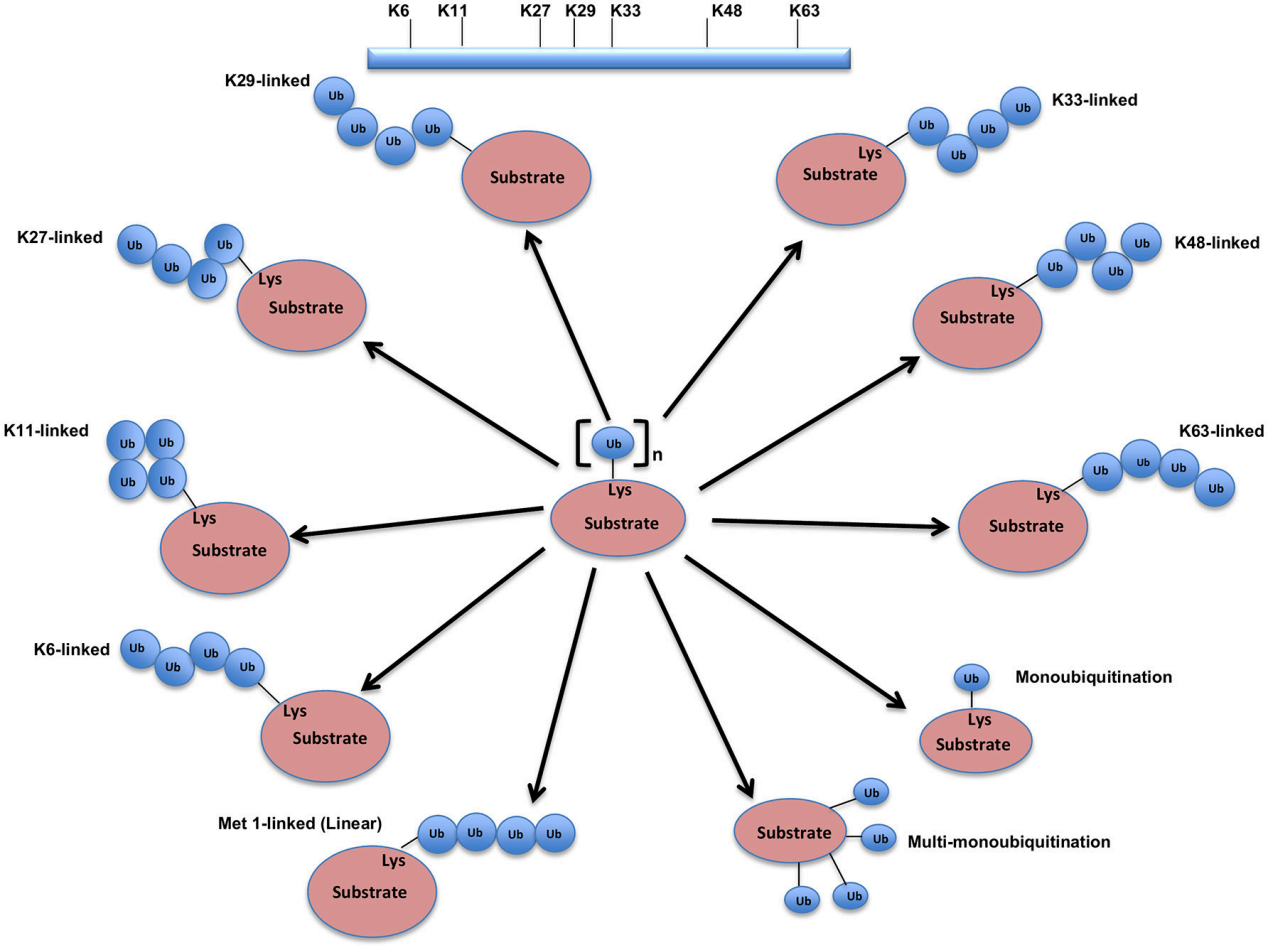
Different types of Ubiquitination and specific function of Ubiquitin linkages. Ubiquitination process can be broken down into three groups, based on how the substrate protein gets tagged by ubiquitin molecule(s). Modification of the substrate with the formation of an isopeptide bond between the C-terminus of the activated ubiquitin and a single substrate lysine leads to the monoubiquitination, when multiple substrates' lysine residues modify with a single ubiquitin molecule each, that results into the multi-monoubiquitination and polyubiquitin chains using one of the seven available lysine residues (K6, K11, K27, K29, K33, K48, or K63) of one ubiquitin molecule attached via an isopeptide bond to the C-terminus of another ubiquitin molecule add further complexity to ubiquitin-encoded protein fate. K48-linked polyubiquitinated proteins are known to be highly unstable and call for degradation via 20S proteasome both for normally folded or misfolded proteins otherwise. K29- and K33-branched mixed chains have shown to regulate AMP-activated protein kinase-related kinases. Polyubiquitination *in vivo* through K29/K33-linked mixed chains blocks their kinase activation by interfering with phosphorylation of the activation-loop residues. K29-branched ubiquitin chains also shown to promote the proteasomal and lysosomal degradation of proteins, whereas K63-branched polyubiquitination majorly plays a key role in various non-degradative processes such as regulation of endocytosis, DNA repair, protein kinase activation, signal transduction, intracellular trafficking of membrane proteins, and stress responses. K63 mediated linkages also known to facilitate the autophagic degradation of substrate proteins and their associated cellular materials, such as damaged mitochondria and invading pathogens. Monoubiquitination and multi-monoubiquitination have been implicated in non-proteasomal regulatory functions like proteins translocation to the nucleus, cytoskeleton and endocytic machinery, virus budding, DNA repair, or modulating enzymatic activity and protein-protein interactions. Majority of the ubiquitin attachment on the protein appears to be at lysine residue, although N-terminal methionine (M1) and cysteine modifications have also been reported. Formation of a peptide bond between the N-terminal methionine residue of one ubiquitin molecule and the C-terminal glycine of the next in the chain results into the linear ubiquitin chains. Linear ubiquitin chains i.e. M1-linked chains primarily play pivotal roles in inflammatory and immune responses. Linear ubiquitin chain is formed by LUBAC (linear ubiquitin chain assembly complex), a multisubunit member of RBR family of E3 ligases. The complex is made of three enzymes: HOIP, HOIL1, and SHARPIN.

The UPS, which represents the major system for extra-lysosomal protein degradation in a cell, confers constitutive and conditional metabolic instability on a protein by temporal control and selective degradation. Here, protein substrates are first tagged through the covalent attachment of single or multiple ubiquitin moieties, and the tagged proteins ultimately get degraded by the 26S proteasome and their component amino acids eventually recycled (Figure 1). Over the years, the general notion of proteasomal degradation has been that of recognition of polyubiquitination chain of four or more ubiquitin attached to substrate protein by the proteasome (Thrower et al., 2000) but increasing number of studies suggests that even monoubiquitination or multi-monoubiquitination (Figure 2) is adequate to degrade small substrates that are longer than 20 amino acids but smaller than 150 residues (Shabek et al., 2007, 2009, 2012; Nakagawa and Nakayama, 2015). Protein ubiquitination and degradation is a three-cascade mechanism as explained in Figure 1.

Recent structural studies reveal two major classes of E3s classified primarily based on the mechanism they follow for transferring ubiquitin from the E2 enzyme onto the substrate. E3s having catalytic HECT (homologous to E6AP carboxyl terminus) domain family creates a catalytic intermediate having ubiquitin attached to its conserved cysteine residue preceding its transfer onto substrate protein (Huibregtse et al., 1995). RING (Really Interesting New Gene)-type and other structurally related ligases constituting the second class of E3s, mediates the direct transfer of ubiquitin from the E2 onto the substrate (Lorick et al., 1999). RING E3s are composed either of single- or multi-subunits. Whereas in single-subunit RING E3s, for instance, MDM2, a single polypeptide itself contains both a catalytic RING finger domain and a substrate interaction domain, the multi-subunit RING E3s perform these functions using separate polypeptides (Petroski and Deshaies, 2005; Eldridge and O'brien, 2010). A common feature of these more complex E3 ligases is the presence of a catalytic core, containing a Cullin family member and a catalytic RING finger protein, which is then targeted to one of many substrates by binding to a substrate-specific adaptor protein. For example, the best-characterized multi-subunit ligase, the SCF complex (Skp1, Cullin, F-box), is recruited to substrates through the adaptor protein Skp1 and an F-box protein substrate receptor binding to one of nearly 70 F-box proteins (Eldridge and O'brien, 2010). The third class of E3 ligases that have been added to the list combines the properties of both RING-type and HECT-type E3 ligases. The RING-between-RING (RBR) E3 ligases consist of two distinct RING domains, called RING1 and RING2, connected by an IBR (In-Between-Ring) domain. While RING1 domain initially recognizes the ubiquitin-loaded E2 (RING-like), RING2 domain offers the active site cysteine residue (HECT-like) which allows it to accept the ubiquitin from the E2 enzyme forming a thioester intermediate and mediates its transfer onto the substrate.

The human genome is known to express nearly 100 DUBs to counterbalance the ubiquitination process. DUBs are classified into either metalloproteases or cysteine proteases, which is further classified into four subclasses of Ubiquitin-specific protease (USP), Otubain protease (OTU), Ubiquitin carboxyl-terminal hydrolase (UCH), and Machado-Joseph disease protease (MJD). The interplay between ubiquitination and deubiquitination sets the threshold for the cellular protein for the proteasomal degradation and has emerged as regulating diverse cellular processes including cell cycle progression and chromosome segregation (Song and Rape, 2008), gene expression (Reyes-Turcu et al., 2009), kinase activation (Komada, 2008), apoptosis (Suzuki et al., 2001; Wilson et al., 2002; Burrows et al., 2004; Shin et al., 2006), localization and degradation of signaling intermediates (Mukhopadhyay and Riezman, 2007; Rytkönen and Holden, 2007) etc.

## Regulation of apoptosis by UPS enzymes

Apoptosis is a major type of modulated cell death process. There are two types of the well-defined apoptotic pathway: intrinsic and extrinsic. These pathways end up in the self-killing process following the activation of caspase cascade. Ubiquitin proteasomal machinery plays a crucial role in regulating apoptosis via targeting important governors of apoptosis and caspases. The interaction between UPS enzymes and apoptotic factors is complex and it can either cause or abolish apoptosis depending upon the signaling factor.

### E3 ligases regulating the intrinsic apoptosis pathway

**Figure 3:**
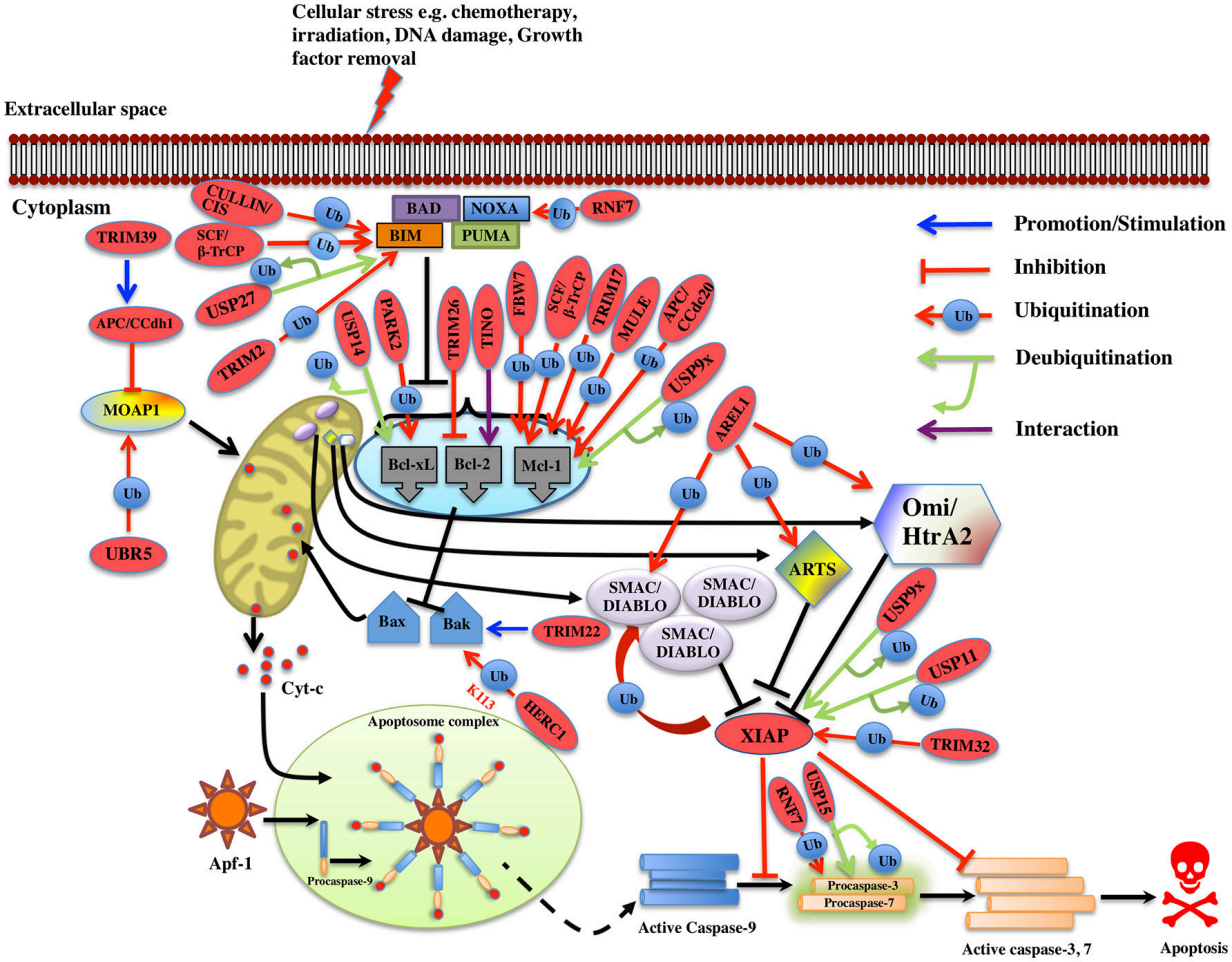
Regulation of Intrinsic pathway by E3 ligases and DUBs. Multiple signals such as DNA damage, unfolded protein response, cytokine deprivation, free radical generating compounds, removal of nutrients, oxygen or growth factors, and endoplasm reticulum stress etc. contributes in the activation of intrinsic or the mitochondrial apoptotic pathway. Following stress, two proapoptotic Bcl-2 (B-cell lymphoma 2) proteins- Bax (Bcl-2 associated X protein) and Bak (Bcl-2 homologous antagonist killer) are activated upon structural changes. They move to the mitochondria, homodimerizes, and generate pores onto the mitochondrial surface, which enhances the membrane permeabilization and cytochrome c then starts to releases. In the presence of ATP, cytochrome c associates with Apaf-1, leading to self-oligomerization of Apaf-1 via CED-4 domains. This leads to the exposure of CARDs of Apaf-1. When these CARDs domain of Apaf-1 and caspase-9 binds, an apoptosome is formed. Dimerization of procaspase-9 forms an active caspase-9, which further activates the caspase-3 and 7 to commit cell death. Bcl-2 family proteins are categorized as antiapoptotic [Bcl-2, Bcl-xl (Bcl-extra-large), Bcl-w, Mcl-1 (Myeloid leukemia cell differentiation 1), Bcl-b and A1], proapoptotic (Bax, Bak, and Bok (Bcl-2 related ovarian killer)] and regulatory BH3-only proteins [Bad (Bcl-2 antagonist of cell death), Bik (Bcl-2-interacting killer), Bid (BH3-interacting domain death agonist), Bim (Bcl-2 interactor mediator of cell death), Bmf (Bcl-2-modifying factor), Noxa, and Puma (p53 upregulated modulator of apoptosis)], Antiapoptotic proteins suppress the apoptotic signals by limiting Bax and Bak thus blocking cytochrome-c release. In case of cellular stress, Bim and Noxa counteract the effect of antiapoptotic proteins. BH3-only proteins release Bax-Bak from inhibition thus promoting Momp (Mitochondrial outer membrane potential) and apoptosis. The IAPs (inhibitors of apoptosis) are the negative regulators of the intrinsic pathway. XIAP (X-linked Inhibitor of Apoptosis Protein) is one of the IAP to be seen upregulated in cancer and is an inhibitor of caspase. Activation of the intrinsic apoptotic pathway leads to the release of Smac/Diablo (Second mitochondrial activator of caspases or direct IAP binding protein with low pI), Omi/HtrA2 (Omi stress-regulated endoprotease or High-temperature requirement A2) and Arts (Apoptosis-related protein in the TGF-beta signaling pathway) into the cytosol. Smac/Diablo and Omi/HtrA2, when released into cytosol upon stimuli, use their IBM (IAP-binding motif) domain to bind to the BIR2 and BIR3 domains of the IAPs. They prevent the XIAPs ability to inhibit caspases and thus mediate apoptosis. Arts interact with residues of the BIR3 domain of XIAP distinct from Smac/Diablo and Omi/HtrA2 suggesting it to antagonize XIAP in a different way. Several E3 ligase and DUBs involved at different stages of the pathway are shown as red ovals.

UPS-mediated regulation of the proapoptotic members of the Bcl-2 superfamily and its outcome on apoptosis are reported for different cell types. Many contradictory results have been reported in different cell types for the role of E3s in the apoptosis. For example, CBL E3 ligase has been identified for BIM splice variant, BimEL (BIM-extra-long) degradation in osteoclasts (Akiyama et al., 2003), however, Wiggins et al showed that CBL is not accountable for the degradation of the same in the fibroblasts and HEK293 cells (Wiggins et al., 2007). Understanding the degradation mechanism of BimEL aims to convey the use of new oncogene-targeted therapeutics. Similarly, the levels of Bim, another proapoptotic protein does not change in the testis of c-CBL knockout mice. Thus, that might prove the role of CBL confined to specific cell types or stimuli (El Chami et al., 2005). Interestingly, Zhang et al. demonstrated BimEL protein degradation by an E3 multisubunit complex consisting of Elongin B/C, Cullin2, and CIS (cytokine-inducible Src homology 2 domain-containing protein), which are SOCS box domain containing E3 ligases. BimEL degradation occurs most likely by means of its bridging with Elongin B/C-Cullin2-CIS ubiquitin-protein isopeptide ligase complex. Further, it was found a RACK1; a 7-WD motif-containing protein plays a vital role in the regulation of BimEL in the presence of the apoptotic agent. The molecular detail of ways RACK1 mediates the formation of this complex is still unclear (Figure 3). These observations support the regulatory roles of CIS E3 ligase in determining BimEL levels and entail its potential role in the development of drug resistance in tumors (Zhang et al., 2008). BimEL activity is regulated via phosphorylation on Ser69 by Erk1/2, which leads to its proteasomal degradation (Luciano et al., 2003). Apparently, phosphorylation of BimEL by Rsk1/2 on Ser93/Ser94/Ser98 facilitates its degradation via its interaction with the F-box proteins β-TrCP1 and β-TrCP2. BimEL was also shown to co-immunoprecipitate with Cullin1 of SCF^β−TrCP1/2^ (Dehan et al., 2009). A recent study showed that Aurora kinase phosphorylates BimEL at Ser93/Ser94/Ser98 consequently targeting it to degradation via β-TrCP1. Many Aurora A inhibitors are in clinical trials as cancer therapeutics (Moustafa-Kamal et al., 2013). A novel ubiquitin-independent pathway has been highlighted for Bim degradation by 20S proteasomes in the absence of poly-ubiquitination proposing it to be an intrinsically disordered protein (Wiggins et al., 2011).

In case of murine B-ALL cells, RING family E3 ligase, TRIM33 prevents apoptosis by interfering with Bim activation. Developing drugs that prevent TRIM33 E3 ligase activity on Bim could offer new options for treating leukemia (Wang et al., 2015). In case of breast cancer, the elevated expression of SIAH1 E3 ligase induces apoptosis by upregulating the expression of Bim via JNK pathway. Conversely, the reduction of SIAH1 in these cells increased the expression of p-ERK and vice versa. Hence, SIAH1 would possibly inhibit the invasive action of cancer cells through ERK pathway (Wen et al., 2010) thus acting as a new therapeutic target in cancer. It has been shown that ubiquitination of Bim is increased following preconditioning ischemia (Meller et al., 2006). To further determine the E3 ligase involved in preconditioning ischemia, a proteomics approach was used and TRIM2 E3 ligase was identified that binds to Bim upon phosphorylation by ERK and leads to Bim ubiquitination. Blocking phosphorylation following preconditioning ischemia reduced the binding between Bim and TRIM2. It was also noted that Bim levels were stabilized upon TRIM2 suppression using shRNA (Thompson et al., 2011). However, whether TRIM2 action on Bim ubiquitination is in conjunction with other proteins or by itself still needs to be determined. It would be interesting to see what conformational changes occur when TRIM2 E3 ligase monomer or dimer interacts with Bim. Additionally, it is far unclear, the type of polyubiquitination linkage regulated by TRIM2. Thus, in-depth studies are required to elucidate the detailed mechanism by which TRIM2 carries out Bim ubiquitination (Figure 3). Another multi-subunit ubiquitin ligase (SAG-SCF^β−TrCP^) containing RING component, SAG also known as RNF7 (RING Finger Protein 7) reduces the Noxa levels when overexpressed; on the contrary, its knockdown delayed the degradation of Noxa. This study recognized Noxa to be a novel substrate of SAG. Another study found that SAG recognizes the first 38 amino acids of procaspase-3 through F-box protein β-TrCP thus promoting the ubiquitination and its degradation (Sun and Li, 2013).

An antiapoptotic protein Mcl-1 that is essential for the survival of multiple cell lineages is tightly regulated under physiological conditions. Recently five E3 ligases, TRIM17, MULE, SCF^β−TrCP^, SCF^FBW7^, and APC/CCdc20 are identified for the polyubiquitination and degradation of Mcl-1 in different cell types. TRIM17 was found to be a critical E3 ligase for the initiation of neuronal apoptosis (Lassot et al., 2010). Overexpression of TRIM17 reduces the protein level of Mcl-1 whereas its knockdown resulted in increased expression and half-life of Mcl-1. Proteasome-dependent degradation of Mcl-1 is proven to be dependent on its prior phosphorylation by GSK3 and failure of which decreased Mcl-1 degradation and disrupted the interaction between TRIM17 and Mcl-1 (Magiera et al., 2013; Figure 3). MULE E3 ligase interacts with Mcl-1 via its BH3 domain leading to its polyubiquitination and degradation. Further, knockdown of MULE stabilizing the Mcl-1 expression was also noticed (Zhong et al., 2005). However, the basal Mcl-1 protein levels are unaffected in Mule-deficient cells (Hao et al., 2012; Inoue et al., 2013). The interaction between MULE and Mcl-1 is found to be weak and can be displaced when Bim and Puma are overexpressed; thus, stabilizing Mcl-1 (Mei et al., 2005; Czabotar et al., 2007; Warr et al., 2011). Other studies showed that binding of Noxa to Mcl-1 triggers its degradation, and favors its interaction with MULE (Willis et al., 2005; Czabotar et al., 2007; Gomez-Bougie et al., 2011). Over-expression of Noxa enhances the MULE/Mcl-1 interaction while reduces the complex formation of MULE with a DUB, USP9X that in the end results in the proteasomal degradation of Mcl-1 (Gomez-Bougie et al., 2011). Structural studies showed that to prompt Mcl-1 degradation, the C-terminal sequence of the Noxa BH3 domain is necessary (Czabotar et al., 2007). GSK3 phosphorylates Mcl-1 at residues Ser155, Ser159, and Thr163 thus associating it with F-box protein β-TrCP (Ding et al., 2007). Suppression of β-TrCP leads to an increase in Mcl-1 levels while its overexpression contributed to Mcl-1 ubiquitination. Akt inhibition and β-TrCP along with FBW7 leads to GSK3-dependent ubiquitination and degradation of Mcl-1 results in apoptosis of lung cancer cells (Ren et al., 2013). In case of both human and mouse the loss of FBW7 resulted in the accumulation of Mcl-1. Reduced Mcl-1 degradation was seen when the interaction between Mcl-1 and FBW7 was impaired due to mutation of the phosphorylation sites Ser159 and Thr163. Mcl-1 degradation is regulated by JNK, p38, CKII, and CDK1 kinases (Inuzuka et al., 2011; Wertz et al., 2011). Clearly, depending upon the cellular context the kinases involved in the phosphorylation of Mcl-1 differs. In addition to SCF^FBW7^, another E3 complex APC/CCdc 20 mediates ubiquitination of Mcl-1 phosphorylated at Thr92 for degradation (Harley et al., 2010; Figure 3).

MOAP1 (Modulator of Apoptosis 1) is a mitochondria effector of Bax, which regulates the release of apoptogenic factors. Its level is maintained in the cell by APC/CCdh1 E3 ligase complex in G1 phase while UBR5 E3 ligase mediates the ubiquitination and degradation of MOAP1 via N-end rule pathway during late S and G2 phase of cell cycle (Huang et al., 2012; Matsuura et al., 2017). There is no lysine residue identified of MOAP1 on which ubiquitination occurs and more experiments need to be accomplished to apprehend the mechanism. However, TRIM39 E3 ligase is known to inhibit the polyubiquitination of MOAP1 suggesting contrasting role. TRIM39 is shown to act on the APC/CCdh1 complex and helps in stabilizing MOAP1. C44A RING domain mutation in TRIM39 is shown to prevent the ubiquitination of MOAP1 by APC/CCdh1, suggesting a role of the RING domain E3 ligase activity of TRIM39 (Huang et al., 2012). It might interact with E1 and E2s involved in MOAP1 degradation pathway and then inhibits the ubiquitination of MOAP1 (Figure 3). The levels of Bak is known to be regulated by HPV E6 recruited HERC1 E3 ligase where it mediates K113-linked ubiquitination and degradation of Bak. The BH3 domain of HERC1 E3 is known to interact with the Bak (Holloway et al., 2015). A more detailed insight is needed on the HPV E6 domain interaction with the HERC1 E3 ligase and at the mechanism of the recruitment whether or not it occurs in response to stress signal or is a condition specific. SOCS6 protein functions as an adaptor protein and links substrate to Elongin B/C-Cullin2/Cullin5 complex via SOCS box and known to induce apoptosis via intrinsic apoptotic pathways. SOCS6 knockdown is shown to result in delayed cytochrome-c release and inhibit apoptosis (Lin et al., 2013). The role of the complex in mediating ubiquitination and degradation of substrates in apoptosis is still not determined. Procaspase-3 is ubiquitinated and gets degraded by SCF^β^^-TrCP^ (Tan et al., 2006). TRIM22 E3 ligase is known to regulate Bak oligomerization and lead to the release of cytochrome-c and caspase-3, 9 activations. It is known that deletion of RING or SPRY domain of TRIM22 decreases the monocyte-mediated apoptosis. Mechanistic details on the Bak oligomerization mediated by TRIM22 and whether it is regulated directly or in the association of other factors need to be further explored as this would provide more understanding on monocyte-mediated apoptosis (Chen et al., 2017). Knockdown of WWP1 E3 ligase is associated with caspase-9 activation, cleavage of PARP and apoptosis. Various studies suggested the role of WWP1 as a biomarker for cancer, however, more studies need to be done in different cancer models (Chen et al., 2007). TRIM9 and ZBTB38 are other E3 ligases where knockdown of these resulted in caspase-9 and 7 activations in human lung carcinoma and C2C12 cells, respectively (Oikawa et al., 2008; Wang et al., 2016). ZBTB38 E3 has SP domain that has caspase recognition sites suggesting it be cleaved by caspase-3 (Oikawa et al., 2008). The existence of two cleavage sites in ZBTB38 protein needs to be further explored and this could entail its role in other biological processes and diseases. AREL1 E3 ligase is identified to mediate degradation of Smac/Diablo, Omi/HtrA2, and ARTS when they are released into cytosol upon apoptotic stimuli (Kim et al., 2013). Identification of the lysine residue that is targeted for ubiquitination of the substrates would provide more insight into the pathway. The antiapoptotic protein XIAP itself has the E3 ligase activity and is seen to overexpress in tumors and considered as a potential drug target. XIAP BIR3 domain heterodimerizes with the caspase-9 monomer and inhibits the homodimerization of caspase-9 (Shiozaki et al., 2003). XIAP also inhibits the caspase-3 and 7, but the mechanism of action was found to be different. XIAP uses its BIR2 domain to bind to caspase-3 and 7 and it inhibits caspases by binding the N terminus of the BIR2 domain (Scott et al., 2005; Figure 3). Recently, TRIM32 is identified either to stimulate XIAP E3 ligase activity or to ubiquitinate and degrade XIAP itself (Ryu et al., 2011). All these findings suggest TRIM32 to be a tumor suppressant gene and further studies could demonstrate the identical function in different tumor models as well.

It is now an established fact that ubiquitin-proteasome system regulates the expression level of many apoptotic proteins. For instance, PHF8 E3 ligase knockdown increases the level of Bax, p21, cleaved caspase-3, and Parp whereas it decreases the expression of antiapoptotic protein Bcl-2 in prostate cancer (Ma et al., 2015). TRIM9 E3 ligase knockdown in human lung cancer tissues and cell lines reduces Bcl-2 expression while it activates the caspase-7 and caspase-9 (Wang et al., 2016). In case of gastric cancer upon cisplatin-induced apoptosis, CAC1 E3 ligase protects the cells by altering Bcl-2*/*Bax ratio. When CAC1 was silenced a pronounced increase of Bax and a decrease in the Bcl-2 level were observed (Zheng et al., 2013). TRIM26 E3 ligase role has been demonstrated in limiting the lung cancer growth, by downregulating Bcl-2 suggesting a potential therapeutic role in cancer treatment (Yi et al., 2016; Figure 3). Further validation of the functional analysis is still required for these E3 ligases in the context of developing potential therapeutic schemes for cancer. E3 ligases are also recognized to regulate the expression of Bcl-2 protein at the transcription level. TINO E3 ligase interacts with the AU-rich element (ARE) of Bcl-2 and has been shown to negatively regulate the Bcl-2 in HeLa cells at transcription level (Donnini et al., 2004; Figure 3; Table 1).

**Table 1:** List illustrating the functions of E3 ligases in regulating intrinsic apoptosis.

**Name**	**Function**
CBL	Degradation of BimEL in cell line-specific manner
Elongin B/C-Cullin2-CIS	Degradation of BimEL
SCF^β^^-TrCP^	Degradation of BimEL and Mcl-1
TRIM33	Activation of Bim
SIAH1	Upregulation of Bim
SAG-SCF^β^^-TrCP^	Ubiquitination and degradation of Noxa and procaspase-3
TRIM2	Ubiquitination and degradation of Bim
TRIM9	Upregulation of Bcl-2 and Downregulation of caspase-9 and 7
CAC1	Alteration of Bcl-2/Bax ratio
TRIM26	Downregulation of Bcl-2
TINO	Downregulation of Bcl-2
TRIM17	Ubiquitination and degradation of Mcl-1
MULE	Ubiquitination and degradation of Mcl-1
SCF^FBW7^	Ubiquitination and degradation of Mcl-1
APC/CCdc20	Ubiquitination and degradation of Mcl-1
APC/CCdh1	Ubiquitination and degradation of MOAP1
UBR5	Ubiquitination and degradation of MOAP1
TRIM39	Stabilization of MOAP1 by inhibiting ubiquitination and degradation of MOAP1
HERC1	Degradation of Bak
SOCS6	Regulation of cytochrome-c release
TRIM22	Release of cytochrome-c and activation of caspase-3 and 9
WWP1	Downregulation of caspase-9 and cleavage of PARP
TRIM9	Downregulation of caspase-9 and 7
WWP2	Downregulation of caspase-7
AREL1	Degradation of Smac/DIABLO, Omi/HtrA2 and ARTS
TRIM32	Degradation of XIAP
TRAF5	Inhibition of Bcl-xL
PRP19	Downregulation of caspase-3, 7 and 9
ZBTB38	Downregulation of caspase-7, 9, 3 but not caspase-8
AREL1	Degradation of Smac/Diablo, Omi/HtrA2 and Arts
XIAP	Degradation of caspase-9, 3 and 7
PARK2	Ubiquitination of Bcl-xL
TRIM32	Ubiquitination and degradation of XIAP

### DUBs regulating the intrinsic apoptosis pathway

Deubiquitination of many intrinsic apoptosis pathway proteins has been reported, where their levels in the cells are maintained by multiple DUBs, however the mechanism of action of these DUBs on the substrate proteins is not very well reported. The expression level of DUBs has been linked to the stabilization or destabilization of apoptotic proteins. In case of Bim protein, its expression is downregulated via ERK-dependent phosphorylation and ubiquitination. USP27x DUB antagonizes the Raf-ERK Bim-degradation pathway thus stabilizing Bim expression. It is also reported that USP27x is upregulated in melanoma and NSCLC cell line suggesting it to act as a tumor suppressor (Weber et al., 2016). Multiple myeloma cells are protected from apoptosis induced by chemotherapy by means of cell adhesion to fibronectin-coated surfaces or bone marrow stromal cells and this is termed as CAM-DR (cell adhesion-mediated drug resistance). USP14 was discovered to negatively regulate cell apoptosis by interacting with Bcl-xl and upregulating its level (Xu et al., 2017). This study suggests USP14 participate in cell adhesion and the inhibitors against USP14 can be applied in therapy, however, the regulatory mechanism is still unknown. In follicular lymphomas, increased expression of Mcl-1 and USP9x is correlated with the involvement of USP9x DUB in deubiquitination and stabilization of Mcl-1 by way of removing K48-linked polyubiquitin chains (Schwickart et al., 2010; Figure 3). Futures studies on the Mcl-1 and USP9x domain will shed more light on their mechanistic details. USP9x is also reported as the mitotic DUB for XIAP and together they are recognized as biomarkers and therapeutic targets for aggressive B cell lymphoma. WP1130 is an inhibitor of USP9x, which is known to upregulate the level of Mcl-1, leading to apoptosis (Engel et al., 2016).

Overexpression of USP15 DUB in paclitaxel-treated HeLa cells is shown to cause deubiquitination of procaspase-3 and its release from Cullin1 of SCF complex, resulting in apoptotic cell death. On the contrary, knockdown of USP15 causes procaspase-3 binding to SCF complex resulting in ubiquitination and degradation and no apoptosis. It is indicated that paclitaxel-resistant tumors occur because of improper functioning of caspase-3 activities (Xu et al., 2009). Knockdown of USP39 has been shown to be associated with increased levels of p21, p27, and Bax, and increased apoptosis (Zhao et al., 2016; Figure 3). Furthermore, a more detailed study in direction of how these proteins depletion leads to apoptosis will provide more insight into the regulation of apoptosis pathway. In breast cancer tissue USP11 is seen to be upregulated and is correlated with the high level of XIAP. C-terminus of USP11 interacts with the BIR2 domain of XIAP and deubiquitinates it leading to inhibition of apoptosis (Zhou et al., 2017; Table 2). The accumulation of XIAP promotes tumor formation and its progression, because of inhibition of apoptosis in cancer cells. However, little is known about the initiation of USP11 accumulation in breast cancer. Future mechanistic studies will help to design inhibitors of USP11 and recognize it as a potential target in breast cancer (Figure 3).

**Table 2:** List illustrating the functions of DUBs in regulating intrinsic apoptosis.

**Name**	**Function**
USP27	Deubiquitination of Bim
USP14	Upregulation of Bcl-xL
USP9x	Deubiquitination of Mcl-1
USP15	Deubiquitination and stabilization of procaspase-3
USP39	Downregulation of p21, p27, and Bax
USP11	Deubiquitination and stabilization of XIAP

### E3 ligase regulating extrinsic apoptosis pathway

**Figure 4:**
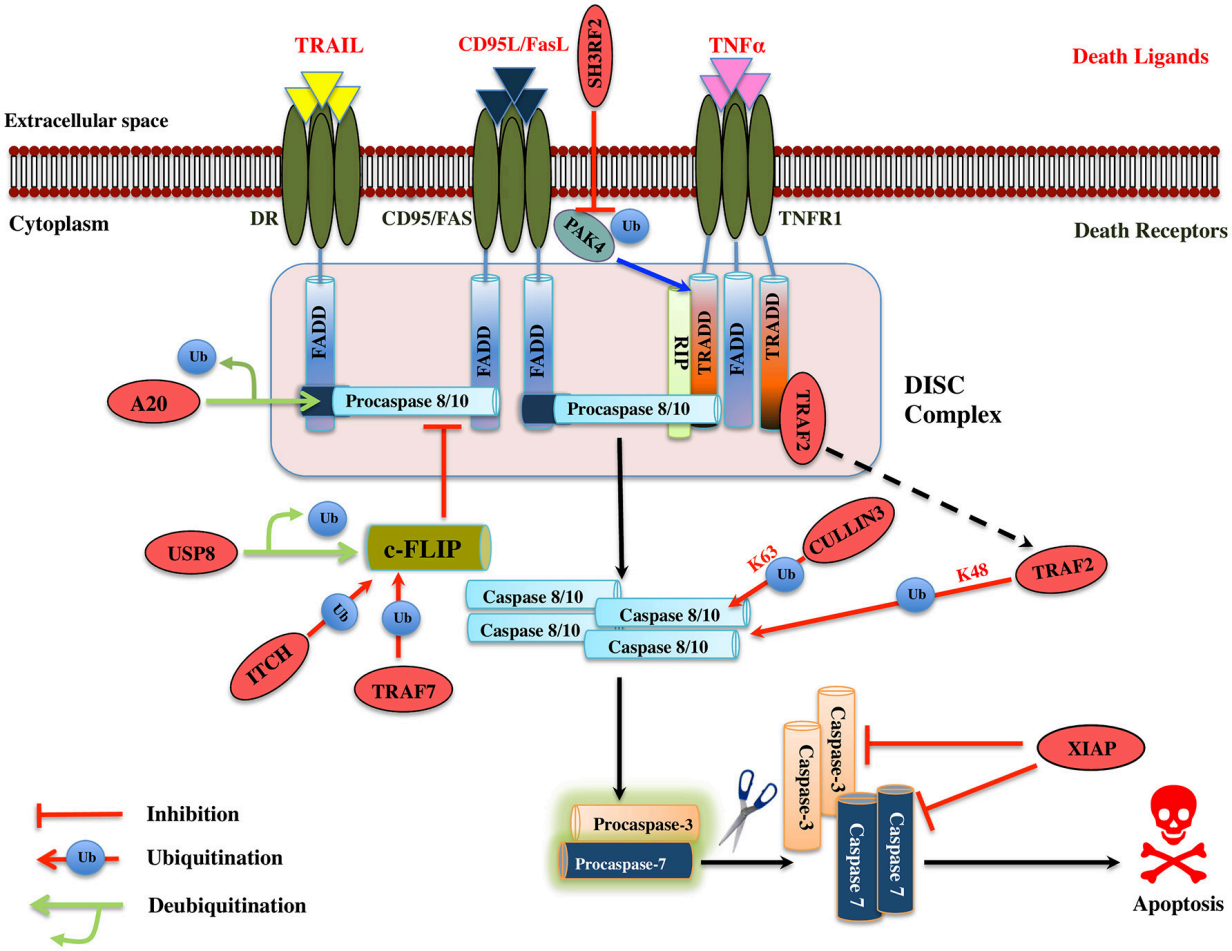
Illustration of E3 ligases and DUBs that regulate extrinsic pathway of Apoptosis. Signaling molecule such as Fas ligand, TNF (Tumor necrosis factor) and TRAIL (TNF related apoptosis-inducing ligand) bind to the transmembrane death receptors like FasR, TNFR, death receptor 4/5, respectively to induce the extrinsic apoptosis pathways. TNF extrinsic apoptosis pathway is initiated by binding of TNF ligand to it two cognate surface receptors, TNF-R1 and TNF-R2. Ligand binding to TNF-R1 results in the dissociation of the SODD (silencer of death domains) from the cytoplasmic tail of TNF-R1 and this facilitates recruitment of adaptor protein TRADD (TNFR1-associated death domain protein). This association further leads to the recruitment of FADD (Fas-associated death domain protein), TRAF2 (TNFR-associated factor 2), TRAF5, RIP (receptor-interacting protein), and c-IAP1/2 (cyto inhibitor of apoptosis proteins) and forms a DISC (death-inducing signaling complex). DISC mediates the enrolment of additional proteins such as the initiator caspase, procaspase-8, which, then proteolytically cleaved, releases an active form of caspase-8, which further activates the effector caspase-3 and 7 and leads to the apoptosis. Activation of caspase-8 is regulated by c-FLIP (cellular Fas-associated death domain-like interleukin 1β-converting enzyme inhibitory protein), a catalytically inactive homolog of caspase 8 that interacts with procaspase-8. Several IAPs (inhibitors of apoptosis) also regulate apoptosis by interacting with TRAF2. Similarly, when Fas protein binds to FasL, it promotes the recruitment of FADD adaptor protein that has DD (Death domain) and DED (Death effector domain) that associate with caspase-8 and 10 and forms a DISC (Hongmei, 2012). Later, apoptosis is preceded just same as in TNF signaling. Several ubiquitin ligases and DUBs that are involved in the pathway and critically regulating the apoptosis are shown as red ovals.

TNF-α plays a role in numerous cellular processes such as differentiation, cell proliferation, and cell apoptosis. The ubiquitin-proteasome machinery regulates TNF mediated apoptosis by controlling levels of many effector proteins in the signaling pathway. PAK4 (p21-activated kinase) is a member of serine-threonine kinases family and it regulates cell survival and apoptosis. Ubiquitinated PAK4 protein is identified to facilitate TRADD recruitment to TNFR1 to form a signal complex and inhibit caspase-8 activation. SH3RF2, a RING E3 ligase prevents the PAK4 ubiquitination in colon cancer (Kim et al., 2013; Figure 4). It would be interesting to know mechanistically how SH3RF2 prevents the ubiquitination of PAK4. The main question to answer is whether SH3RF2 prevents the association of PAK4 with other E3 ligase or factors responsible for PAK4 ubiquitination. TRADD further recruits DISC proteins such as FADD, TRAF5, TRAF2, RIP, and c-IAP1/2, which are regulated by ubiquitin-proteasome players. HACE1, a HECT domain E3 ligase mediates K63-linked polyubiquitination of TRAF2, leading to activation of NF-κB and JNK pathways. Further, HAC1 knockdown impairs the association of TRAF 2 with FADD, which prevents apoptosis, highlighting the role of HACE1 E3 ligase as a tumor suppressor (Tortola et al., 2016; Figure 4). c-FLIP protein interferes with the caspase-8 activation at the death receptor-mediated apoptosis; the level of this protein is upregulated in multiple forms of cancers. TRAF7 itself a RING E3 ligase promotes the K29-linked polyubiquitination of c-FLIP thus maintaining its turnover by the lysosomal pathway. Interestingly, TRAF7 also activates the NF-κB, which then activates the c-FLIP in a contradictory way (Zotti et al., 2011; Scudiero et al., 2012). The dual role played by TRAF7 could be a signal dependent but future mechanistic studies on TRAF7 will provide more insight into its biological function. ITCH, another HECT domain E3 ligase is known to ubiquitinate and degrade c-FLIP_L_ by interacting with CASP domain, thus inhibit apoptosis by preventing caspase-8 activation (Chang et al., 2006). ITCH is first activated by JNK pathway and degrades c-FLIP, suggesting the role of JNK in mediating the TNF induced apoptosis (Chang et al., 2006; Figure 4). A multisubunit E3 ligase complex (Rbx1-Cullin 3) is identified to induce polyubiquitination of caspase-8 at K461 leading to the recruitment of Ub-binding protein p62 that follows its aggregation and release into the cytosol where it mediates the activation of other downstream caspases hence apoptosis (Jin et al., 2009). Another RING E3 ligase TRAF2 mediates the K48-linked polyubiquitination of caspase-8 and is suggested to control the levels of the same (Gonzalvez et al., 2012; Figure 4). However, the role of TRAF2 as an E3 ligase is questionable and more proofs are needed to understand the mechanistic of its E3 ligase activity. XIAP is known to inhibit both the caspase-3 and 7. ZBTB7A, BTB domain containing E3 ligase is overexpressed in hepatocellular carcinomas and silencing of this gene leads to Fas and FADD expression (Zhang et al., 2013; Figure 4; Table 3). This suggests it could be a potential drug target in cancer therapy if the further role of this protein is studied in different cancer cell line models.

**Table 3:** List mentioning the functions of E3 ligases involved in TNF induced apoptosis regulation.

**Name**	**Function**
SH3RF2	Inhibition of ubiquitination of PAK4
HACE1	K63-linked polyubiquitination of TRAF2 thus activation of NF-κB and JNK-mediated apoptosis
TRAF7	Degradation of c-FLIP
Rbx1-Cullin3	Polyubiquitination and activation of caspase-8
TRAF2	K48-linked polyubiquitination of caspase-8
ITCH	Degradation of c-FLIP_L_
XIAP	Degradation of caspase-3 and 7
ZBTB7A	Inhibition of Fas-mediated apoptosis

### DUBs regulating extrinsic apoptosis pathway

A20 has a dual regulatory function of deubiquitination and ubiquitination played by its OTU DUB domain and C_2_/C_2_ ZnF E3 ligase motif, respectively. A20 deubiquitinates procaspase-8, which inhibits its aggregation, consequently inhibits apoptosis (Jin et al., 2009; Figure 4). Whether or not A20 can also work, as E3 ligase for caspase-8 is a case of a future investigation. Caspase-10 is structurally similar to caspase-8; however, no such polyubiquitination has been observed in it, suggesting us to mechanistically consider the polyubiquitination in caspases carefully. USP8 DUB prevents c-FLIP_L_, degradation and further halts the apoptosis in cancer cells suggesting it to be a potential drug target (Panner et al., 2010; Table 4). Future studies ought to pave the way for the recognition of additional DUBs for the extrinsic apoptosis pathway substrate protein such as TRADD, FADD, TRAF2, and TRAF5.

**Table 4:** List mentioning the functions of DUBs involved in TNF induced apoptosis regulation.

**Name**	**Function**
A20	Deubiquitination of procaspase-8
USP8	Deubiquitination and stabilization of c-FLIP_L_

### E3 ligase regulating TNF induced NF-κB signaling

**Figure 5:**
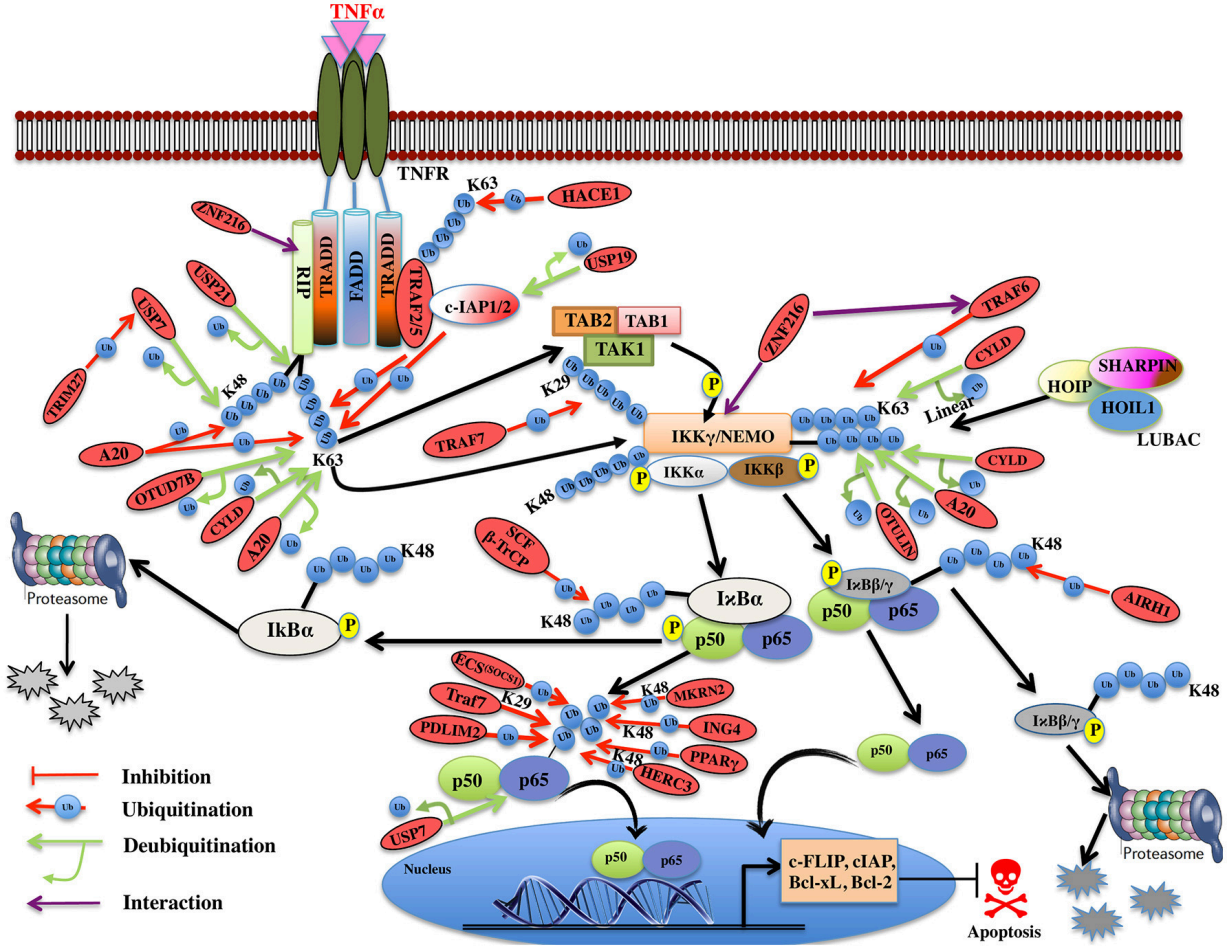
Illustration of E3 ligases and DUBs that regulate TNF induced NF-κB pathway. TNFR signaling also results in the activation of cell survival via activation of NF-kB pathway by recruiting TRADD, TRAF1, and TRAF2, which further enroll RIP1 and c-IAP1/2. TRAF2 is very pivotal in activating the IKK complex (IKKα and IKKβ) and recruits it to the TNF-R1 complex upon phosphorylation of these proteins by TAK2/3 proteins of TAK1 kinase complex. IKKα and IKKβ kinases, phosphorylate Ser32 and Ser36 in IκB isoforms (IκBα, IκBβ) that leads to their ubiquitin-dependent degradation and entry of NF-κB transcription factors p50, p65 (RelA) heterodimer into the nucleus upon activation leading to transcription of genes such as c-FLIP, c-IAP, Bcl-xL, Bcl-2 etc. Red oval indicates the E3 ligases and DUBs involved at various stages.

NF-κB assumes a crucial role in cell survival, inflammation, and cell apoptosis. It is generally considered as antiapoptotic but in certain cellular stresses also has a proapoptotic role. Ubiquitination is one of the very important post-translational modifications in the TNF mediated NF-κB signaling and it helps in determining the protein fate via different ubiquitin linkages. TRAF family proteins function both as adaptor proteins and E3 ligases in cell signaling. The general structure of TRAF protein has C-terminal TRAF domain, which is composed of the coiled-coil region followed by β-sandwich besides TRAF7 (Park et al., 1999). Except for TRAF1, all of them have an N-terminal RING domain and variable zinc fingers that contains the core ubiquitin ligase catalytic domain (Xie, 2013). TRAF2 has the RING E3 ligase activity and is known to polyubiquitinate RIP1 at K63, in coordination with a UBC13/UEV1 E2 enzyme (Yeh et al., 1997; Tada et al., 2001; Shi and Kehrl, 2003; Lee et al., 2004). Interaction of RIP1 with TRAF2 occurs via β-sandwich region in TRAF domain. It was assumed TRAF2 may act like TRAF6 in mediating K63-linked polyubiquitination, but the RING structure of TRAF2 is distinct from TRAF6. The role of TRAF2 as an E3 ligase is controversial as there are structural variations with respect to TRAF6 E3 ligase and differences at the E2 interacting site (Yin et al., 2009; Figure 5). Interestingly, TRAF2 is also known to recruit c-IAP1/2 (cellular inhibitor of apoptosis), the antiapoptotic proteins working as E3s that mediates K63-linked polyubiquitination of RIP1 (Mahoney et al., 2008), therefore, promotes NF-kB activation and block apoptosis (Bertrand et al., 2008; Varfolomeev et al., 2008). Whether TRAF2 just recruits and regulates c-IAP1/2 E3 ligase activity or also has a role in ubiquitination is a matter of further investigation. The specific roles, conditions, and triggers of TRAF2, c-IAP1/2 role in RIP1 ubiquitination remain unknown and await more clarification. NF-kB activation by c-IAP1/2 also needs to be elucidated. A20 by its ZnF E3 ligase domain mediates the K63-linked polyubiquitination of RIP1, which then attaches to caspase-8, blocking caspase-8 dimerization and averts apoptosis (Bellail et al., 2012). A20 is also known to mediate K48-linked polyubiquitination of RIP1 (Wertz et al., 2004; Figure 5).

Recently, an M1-linked ubiquitin chain was identified in NEMO that is mediated by LUBAC (Linear Ubiquitin Chain Assembly Complex), which is formed by HOIL1 (Heme-oxidized IRP2 ubiquitin ligase 1), HOIP1 (HOIL1-interacting protein), and SHARPIN (SHANK-associated RH domain-interacting protein) proteins (Kirisako et al., 2006; Tokunaga et al., 2009; Kensche et al., 2012). NEMO is also K63-linked polyubiquitinated by TRAF6 E3 ligase utilizing Ubc13 as E2 and resulted in the recruitment of TAK1, which is associated with TAB1/2 and IKK (Lamothe et al., 2006). It will be interesting to see how these two modifications collectively modulate the function of NEMO. IκBα is K48 ubiquitinated by ROC1-SCF^β^^-TrCP^ multisubunit E3 ligase complex whereas IκBβ by the aid of ARIH1 E3 ligase, leading to degradation of both the subunits (von Stechow et al., 2015; Figure 5). p65 levels have been known to be regulated by multiple E3 ligases. TRAF7 E3 ligase polyubiquitinates p65 and NEMO at K29 and targets them for lysosomal degradation leading to cell death (Zotti et al., 2011). Some other proteasomal independent mechanisms regulating the level of these proteins have also been proposed and this needs to be further explored. p65 is phosphorylated at Ser276 and Ser536 and nine other different sites, which further regulate its ubiquitination, via E3 ligase complex ECS^SOCS1^ (Elongin B/C-Cullin2/Cullin5-SOCS1) linked to Rbx1 (Cai and Yang, 2016; Christian et al., 2016). COMMD1 as a cofactor and GCN5 (histone acetyltransferase) are known to interact with this complex independently and promote p65 ubiquitination (Mao et al., 2009). PDLIM2 and MKRN2, E3 ligases are also known to bind and promote ubiquitination of p65 independently (Shin et al., 2017). Another E3 ligase ING4 uses its PHD domain for K48-linked polyubiquitination and degradation of p65 using E2 enzyme UbcH3 (Hou et al., 2014). Although the polyubiquitin linked lysine site has not been identified in p65 for any of the E3 ligases discussed above, Lys28 in p65 was identified to be K48-linked polyubiquitinated by E3 ligase PPARγ for the first time (Hou et al., 2012; Figure 5). HERC3, a cytoplasmic E3 ligase is known to mediate K48 polyubiquitination of p65 at two major sites Lys195 and Lys315 before it enters the nucleus (Hochrainer et al., 2015; Figure 5). There are several E3 ligases controlling the degradation of p65, at the same time, it is not clear whether they are working independently or cooperatively to regulate the level of p65, or the levels are maintained by distinct E3 ligases in the event of different stimuli or as such in different cells. Future studies in this pathway would offer more insight into the p65 regulation by different E3 ligases. IκBα factor is maintained by the TRIM13 downregulation, which further induces apoptosis in multiple myeloma (Gatt et al., 2013). This study highlights the role of TRIM13 as a potential drug target, however, more substrate proteins, which are modulated by TRIM13 in NF-κB pathway, needs to be investigated. Further, ZNF216 that contains A20-like zinc finger domain at its N-terminal is known to interact with IKKγ, RIP, and ZnF-AN1 domain. ZNF216 interacts with TRAF6 through its C-terminal consequently resulting in inhibition of the NF-κB activation and antiapoptotic genes activation. Overexpression of ZNF216 sensitizes cells to TNF-induced apoptosis (Huang et al., 2004; Table 5). This study suggests a distinct role of ZNF216 from A20, which inhibits TNF-induced apoptosis (Figure 5). However, more study needs to be done to comprehend mechanistic detail concerning its E3 ligase activity.

**Table 5:** List exhibiting the functions of E3s involved in TNF induced NF-κB regulation.

**Name**	**Function**
TRAF2	K63-linked polyubiquitination of RIP1
c-IAP1/2	K63-linked polyubiquitination of RIP1
A20	K63-linked polyubiquitination of RIP1
A20	K48-linked polyubiquitination of RIP1
LUBAC	M1-linked ubiquitination of NEMO
ARIH1	K48-linked polyubiquitination of IκBβ
SCF^β^^-TrCP^	K48-linked polyubiquitination of IκBα
TRAF7	K29-linked polyubiquitination of p65 and NEMO
ECS^SOCS1^	Polyubiquitination and degradation of p65
PDLIM2	Polyubiquitination and degradation of p65
MKRN2	Polyubiquitination and degradation of p65
ING4	Polyubiquitination and degradation of p65
PPARγ	K48-linked polyubiquitination of p65
HERC3	K48-linked polyubiquitination of p65
ZNF216	Interacts with IKKγ, RIP and ZnF-AN1 domain and inhibits NF-κB activation
TRIM13	Downregulation of IκBα
HACE1	K63-linked polyubiquitination of TRAF2

### DUBs regulating TNF induced NF-κB signaling

DUBs A20, OTUD7B, and CYLD are responsible for the removal of K63-linked polyubiquitin chain from RIP1 (Kovalenko et al., 2003; Enesa et al., 2008; Bellail et al., 2012). A20 prevents linear ubiquitination by way of binding to linear polyubiquitin chains; prevents interaction between NEMO and LUBAC, which further inhibits NF-κB activation (Verhelst et al., 2012). CYLD and OTULIN interact with the LUBAC via HOIP and cleave linear ubiquitin chain from NEMO (Elliott et al., 2014; Draber et al., 2015). CYLD is also known to remove K63-linked polyubiquitin chain from NEMO. This results in inhibition of NF-κB activation and loss of CYLD is shown to inhibit apoptosis (Trompouki et al., 2003; Figure 5). A20 cooperates with other proteins to inhibit the NF-κB signaling and is termed as the A20 ubiquitin-editing complex. TAX1BP1 (Tax1-binding protein 1) cooperates with A20 and interacts with RIP1, which further inhibits the NF-κB activation by recruiting Itch, a HECT E3 ligase. Itch and TAX1BP1 further promote RIP1 degradation by Itch interacting with another E3 ligase RNF11 (Kitching et al., 2003; Shembade et al., 2007, 2008; Shembade and Harhaj, 2012). The mechanistic details of these interactions and deubiquitination are still not clear and more study in this direction needs to be done. USP21 is also identified to promote deubiquitination of both K48- and K63-linked polyubiquitinated RIP1 but whether USP21 functions in coordination with the above-mentioned E3s needs to be elucidated (Xu G. et al., 2010). USP7 also deubiquitinates RIP1 following activation by the TRIM27 E3 ligase resulting in apoptosis (Figure 5). USP7 can remove K27-, K29-, and K48-linked polyubiquitin from RIP1 however not K63-linked polyubiquitin chain (Zaman et al., 2013; Figure 5). This study has highlighted new E3-DUB interaction and similar interaction can likewise be looked in case of linear ubiquitination. Future studies focusing on how the other identified E3 ligases and DUBs together modulate the ubiquitination and deubiquitination process of RIP1 needs to be addressed. c-IAP, an antiapoptotic protein having E3 ligase activity is also known to been regulated by DUBs. USP19 is shown to prevent ubiquitination of c-IAP1/2 and stabilizes them (Mei et al., 2011). There are two distinctively conceivable mechanisms that are considered: either it prevents the binding of E2 to the E3 ligase or it will mask the lysine residue for ubiquitination. The exact mechanism of action for USP19 will provide more insights on how c-IAP1/2 levels are maintained in the cell. USP7 identified as DUB for p65, maintained the balance between ubiquitination and deubiquitination (Colleran et al., 2013; Table 6). The study spotlights USP7 as the potential target for inhibiting NF-κB pathway at the level of the gene promoter. Future studies in the identification of ubiquitination site of p65 will enlighten the role of USP7.

**Table 6:** List exhibiting the functions of DUBs involved in TNF induced NF-κB regulation.

**Name**	**Function**
A20	Deubiquitination of K63-linked polyubiquitin chain in RIP1, Deubiquitination of M-1 linked polyubiquitin chain in NEMO
CYLD	Deubiquitination of K63-linked polyubiquitin chain in RIP1, Deubiquitination of M-1 and K63-linked ubiquitin chain in NEMO
OTUD7B	Deubiquitination of K63-linked polyubiquitin chain in RIP1
OTULIN	Deubiquitination of linear ubiquitin chain in NEMO
USP21	Deubiquitination of RIP1
USP19	Deubiquitination of c-IAP1/2
USP7	Deubiquitination of RIP1 and p65

## p53-mediated apoptosis

p53 is a transcription factor capable of controlling the expression of genes engaged in various cellular processes like apoptosis, growth arrest, DNA repair, autophagy and senescence in response to stress signals (Vogelstein et al., 2000). Upon DNA damage p53 induces the upregulation of TNFR1, DR5 (Death Receptor 5), and Fas mRNAs thus promoting cell death through the activation of caspase-8 (O'Connor et al., 2000; Liu et al., 2004). It also induces the activation of the Bax, Puma, and Noxa proteins causing mitochondrial pore formation and release of cytochrome-c (Oda et al., 2000; Nakano and Vousden, 2001). p53 acts directly on mitochondria to induce apoptosis. The localization of p53 to the mitochondria in response to stress is carried out by mitochondrial-import peptides to promote the oligomerization of Bax and Bak, which then counteracts the effect of Bcl-2 and Bcl-xL. This leads to the mitochondrial permeability and release of apoptogenic factors (Schuler et al., 2000; Vaseva and Moll, 2009). The activity of p53 is tightly modulated by ubiquitination, phosphorylation, sumoylation, acetylation, and methylation (Appella and Anderson, 2001). Ubiquitination process is dynamic, reversible, and is critical for regulating p53 levels under various conditions (Brooks and Gu, 2011; Hock and Vousden, 2014). Since the apoptotic function of p53 plays a prominent role in tumor suppression, understanding the modulation of p53 by ubiquitin machinery might offer novel therapeutic approaches for the treatment of cancer.

### E3 ligases regulating p53-mediated apoptosis

**Figure 6:**
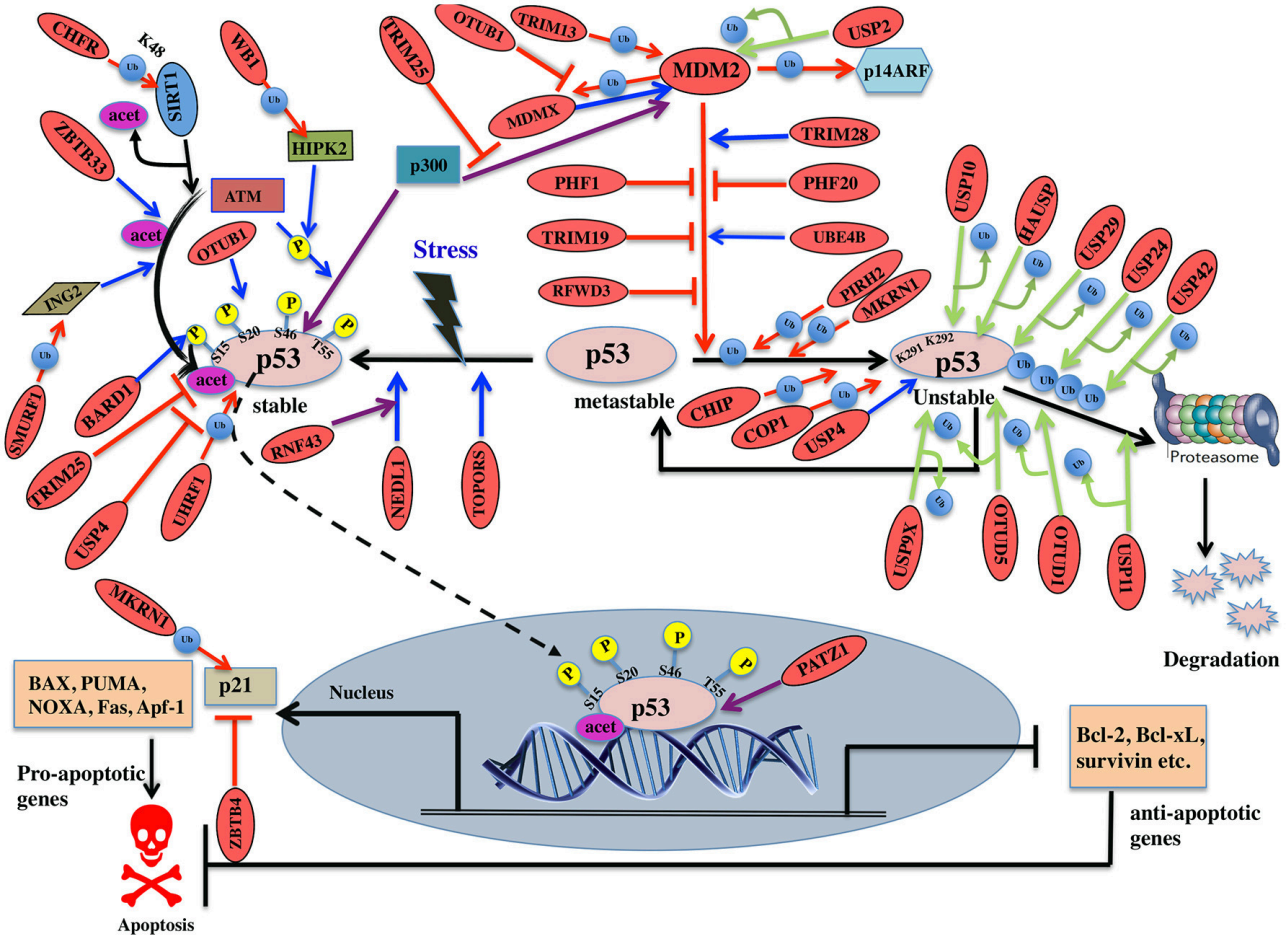
Pictorial representation of p53 pathway regulated by E3 ligases and DUBs. p53 is a transcription factor involved in apoptosis in response to stress signals. p53-p300 interaction is inhibited by MDM2, thus reducing p53 activity. p53-mediated apoptosis signaling is regulated by many E3 ligases and DUBs shown as red ovals.

Under the normal condition, p53 is tightly controlled by an E3 ligase MDM2 that mediates its ubiquitination and degradation through the proteasomal system (Haupt et al., 1997; Kubbutat et al., 1997; Momand et al., 2000). Binding of N-terminus of MDM2 to p53 obstructs its transcriptional activities. Also, the C-terminal RING domain enables MDM2 to recruit E2 ubiquitin-conjugating enzyme, which facilitates p53 ubiquitination and degradation (Poyurovsky et al., 2007). For ubiquitination, MDM2 is known to target six lysine residues on p53 at positions 370, 372, 373, 381, 382, and 386 (Rodriguez et al., 2000). It was noted in mouse embryonic fibroblasts that mutant p53 (p53R172P) is also a substrate of MDM2. In addition to this, mutant p53 is also targeted by CHIP E3 ligase (Lukashchuk and Vousden, 2007; Figure 6). MDMX (MDM4) has structural similarity with MDM2 and interacts with N-terminal transactivation domain of p53 (Shvarts et al., 1996). It oligomerizes via RING domain with MDM2 and increases its efficiency (Wade et al., 2010; Wang et al., 2011). Therefore, the regulation between MDM2 and MDM4 ascertains a correct level and activity of p53. ARF (Alternative Reading Frame) is an important tumor suppressor and known to suppress unusual cell growth in response to oncogenic stimuli. MDM2 interacts with p14ARF, which leads to its degradation, thereby resulting in stabilization and reactivation of p53 protein (Zhang et al., 1998). Another E3 ligase involved in degradation of p53 is ARF-BP1 (MULE), a HECT containing E3 ligase. Its inactivation encourages tumor suppression effects in both p53 null and wild-type cells (Chen et al., 2005; Figure 6).

Several E3 ligases from TRIM family serve to modulate MDM2. TRIM28 through its coiled-coil domain binds to MDM2 and promotes p53 degradation. Silencing of TRIM28 induces DNA damage response and apoptosis and promotes p53 transcriptional activity (Wang et al., 2005). TRIM13 another E3 ligase forms a complex with MDM2 and contributes to the degradation of MDM2 both *in vitro* and *in vivo* (Joo et al., 2011). Similarly, TRIM19 or PML binds to MDM2 causing stabilization of p53 (Bernardi et al., 2004). In addition to the above interactions, cellular level of MDM2 also affects p53 ubiquitination. Low levels of MDM2 monoubiquitinates p53 while a high-level leads to its polyubiquitination (Li et al., 2003; Figure 6). It is known that monoubiquitinated p53 moves to the cytoplasm and recruits E4 enzymes which then binds to the oligo-ubiquitylated substrates and in coordination with E1, E2, and E3, catalyzes the multi-ubiquitination and degradation of p53 (Koegl et al., 1999). In normal cells, p300/CREB (cAMP-response element-binding protein) have both E3 and E4 activities for p53 polyubiquitination and degradation (Shi et al., 2009). Similarly, UBE4B having E3 and E4 ligase activities binds to p53 and MDM2 and leads to inhibition of p53 dependent apoptosis (Wu et al., 2011).

PHF20 a subunit of lysine acetyltransferase binds to p53 with its Tudor domain and dimethylated at two sites; Lys370 or Lys382 thus preventing MDM2-mediated ubiquitination (Cui et al., 2012). The binding site of PHF20 overlaps with the p53 ubiquitination sites, possibly contributing to the stability of p53. E3 ligases PHF1 and RFWD3 prevent complex formation between MDM2 and p53 thus promoting the stability of p53 (Fu et al., 2010; Yang et al., 2013). *In vitro* experiments showed that MDM2 via its acidic domain forms a complex with RFWD3 and restricts the polyubiquitination of p53 (Fu et al., 2010). In addition to MDM2, p53 ubiquitination is carried out by a number of other ubiquitin E3 ligases. PIRH2 and COP1 are among such ligases, which interact with p53 and mediate its ubiquitination and degradation (Leng et al., 2003; Dornan et al., 2004; Figure 6). It has also been shown that MDM2, MDM4, PIRH2, and COP1 might act as a negative regulator of p53 in a synergistic manner (Wang et al., 2011). TOPORS has been shown to act as dual SUMO-1 and RING domain dependent E3 ligase for p53 (Rajendra et al., 2004; Weger et al., 2005). CARPs (Caspase-8/10-associated RING proteins) targets phosphorylated p53 at Ser20 for the degradation. In addition, other E3 ligases such as TRAF7 and Cullin4B catalyze the interaction between ubiquitin and p53 (Wang et al., 2013; Thirunavukarasou et al., 2014). Therefore, they have also been identified as negative-feedback regulators due to their ability to downregulate p53 stability (Figure 6). Thus, modulation of ubiquitinated p53 is an important area for future anti-cancer research and therapeutic development. Literature proposes that apart from the known six C-terminal lysines, there are other sites for ubiquitination and degradation of p53. Under normal conditions, MKRN1 interacts with the core domain of p53 and downregulate p53 and p21 level by ubiquitin-dependent degradation (Figure 6). Lys291 and Lys292 sites are required here to mediate this polyubiquitination and degradation. While in stress conditions, MKRN1 prevents the accumulation of p21 and doesn't affect the p53 levels. This study showed that uninterrupted disruption of p21 by MKRN1 seems to be important for cells to become more prone to apoptosis (Lee et al., 2009). In cancerous cells, NEDL1; an E3 ligase binds to C-terminus of p53 and contributes to proapoptotic functions (Li et al., 2008; Figure 6). In case of colorectal carcinogenesis, another E3 ligase RNF43 was shown to interact with NEDL1 thus inhibiting p53-mediated transcription and apoptosis upon UV induction (Shinada et al., 2011). However, these findings didn't clarify whether this interaction contributes to posttranslational modifications of p53.

During stress, ATM kinase triggers p300 binding to p53 by phosphorylating at Ser15 while it promotes dissociation of MDM2 and p53 by phosphorylating at Ser20. Studies have suggested BARD1, an E3 ligase is involved in phosphorylation of p53 at Ser15 (Feki et al., 2005). p300 also acetylates p53 and an E3 ligase ZBTB33 modulates this acetylation, which thereby contributes to increased expression of apoptotic genes (Koh et al., 2014). ING2 and HIPK2 are the tumor suppressor genes identified to activate p53 by enhancing acetylation at Lys302 and phosphorylation of p53 at Ser46, respectively. WD40 repeats of WSB-1 E3 ligase recognizes HIPK2 and its SOCS box polyubiquitinates and degrades HIPK2 (Choi et al., 2008). SMURF1, a HECT E3 ligase has been shown to interact with ING2 both *in vivo* and *in vitro*. It interacts with the central region of ING2 and targets the PHD domain for poly-ubiquitination and proteasomal degradation (Nie et al., 2010; Figure 6). Downregulation of TRIM25 E3 ligase has been shown to increase acetylation of p53 and cell death. It reduces p53 polyubiquitination by hindering the binding of p300 to MDM2 (Zhang et al., 2015). Another E3 ligase, UHRF1 has been identified to promote polyubiquitination of p53 but does not lead to its degradation (Ma et al., 2015). Detailed mechanism and functional impact of UHRF1-mediated p53 ubiquitination are yet to be determined which would help in recognizing UHRF1 as a therapeutic drug target.

SIRT1, a deacetylase, removes acetyl groups from p53 during DNA damage. SIRT1 is bound to H3K9, which results in decreased H3K9 acetylation and increased trimethylation. The stability and function of SIRT1 are further controlled by CHFR, a RING-type E3 ligase that interacts with and destabilizes SIRT1 by K48-linked ubiquitination and subsequent proteolysis (Kim et al., 2016; Figure 6). p53 transcriptionally activates the expression of p21 gene, which further regulates cell cycle (Figure 6). ZBTB4, a POZ domain zinc-finger protein forms a complex with Miz1 (ZBTB17) a transcription factor and Sin3, which is a histone deacetylase co-repressor and represses the expression of p21 (Weber et al., 2008). p53 initiates apoptosis by transactivating Bax, Bid, Noxa, and Puma and antagonizes the function of Bcl2 and Bcl-xL. Depending on cellular context, PATZ1 a member of POZ and kruppel-like zinc finger family is known to interact with p53 and enhance p53 regulated gene expression. Conversely, in the absence of p53, PATZ1 enhances cell survival (Valentino et al., 2013). This dual role was shown in human embryonic kidney cells and osteosarcoma cells (Table 7).

**Table 7:** List exhibiting the functions of E3 ligases involved in p53-mediated apoptosis.

**Name**	**Function**
MDM2	Ubiquitination and degradation of p53
MDMX	Stabilization of MDM2 and increases its efficiency
ARF-BP1/MULE	Ubiquitination and degradation of p53
TRIM28	Ubiquitination and degradation of p53
TRIM13	Degradation of MDM2
TRIM19	Stabilization of p53 by binding to MDM2
UBE4B	Inhibition of p53 dependent apoptosis
PHF20	Stabilization and activation of p53
RFWD3	Stabilization of p53 by binding to MDM2
PHF1	Stabilization of p53
PIRH2	MDM2-independent ubiquitination and degradation of p53
COP1	Ubiquitination and degradation of p53
TRAF7	Ubiquitination and degradation of p53
CULLIN4B	Ubiquitination and degradation of p53
MKRN1	Ubiquitination and degradation of p53 and p21
NEDL1	Stabilization and activation of p53
BARD1	Involvement in phosphorylation of p53
ZBTB33	Modulation of acetylation of p53
WSB-1	Ubiquitination and degradation of HIPK2
SMURF1	Ubiquitination and degradation of ING2
TRIM25	Inhibition of p53 polyubiquitination
UHRF1	Polyubiquitination of p53
CHFR	Ubiquitination and degradation of SIRT1
ZBTB4	Inhibition of p21
PATZ1	Upregulation of p53 regulated gene expression
TOPORS	Ubiquitination and degradation of p53
CHIP	Ubiquitination and degradation of p53

### DUBs regulating p53-mediated apoptosis

Deubiquitination is an important mechanism known to modulate p53. A number of DUBs have been reported to either upregulate or downregulate p53 dependent signaling. Studying the interaction between DUBs and p53 can recognize potential candidates for cancer therapeutics. A DUB named USP7/HAUSP has a dual function in deubiquitinating and stabilizing both MDM2 and p53 proteins (Sheng et al., 2006). Structural studies showed that HAUSP's N-terminal TRAF-like domain is recognized by both p53 and MDM2. MDM2 makes more conserved interactions with HAUSP as compared to p53 (Hu et al., 2006). Experimental studies showed that the complete loss of HAUSP leads to p53 induced apoptosis and its partial impairment exhibits p53 destabilization. USP7 is also required for the stability of MDMX (Brooks et al., 2007). Another DUB, USP10 deubiquitinates p53, but not MDM2 or MDMX. Under stress condition, ATM phosphorylates USP10, which then deubiquitinates and targets cytoplasmic p53 back to the nucleus. USP10 is reported to be downregulated in renal clear cell carcinomas suggesting it may have tumor suppressive functions (Yuan et al., 2010). USP11 also deubiquitinates and stabilizes p53 in response to DNA damage. Its overexpression activates p53 and elevates the expression of p53 target genes like p21, Puma, and Bax (Ke et al., 2014; Figure 6). USP2 deubiquitinates and stabilizes MDM2 thus inhibiting the proapoptotic activity of p53. In this way, it could possibly contribute to therapeutic resistance in CTCL (cutaneous T-cell lymphomas; Wei et al., 2016). Under both normal and stressed conditions p53 is also deubiquitinated by USP24 and thus stabilizing its level. It has been shown that USP24 targets p53 to regulate UV induced apoptosis (Zhang and Gong, 2016). Future mechanistic understanding will provide insight for its use as cancer therapeutic.

Another DUB, USP4 deubiquitinates HDAC2 (Histone deacetylases 2) that was shown to interact with p53 thereby inhibiting the level of acetylated p53 (Li et al., 2016; Figure 6). Thus, it was hypothesized that USP4 targets acetylated p53 by stabilizing HDAC2. USP4 negatively regulates p53 by binding to ARF-BP1 and deubiquitinates it thereby promoting ARF-BP1-dependent ubiquitination and degradation of p53 (Zhang et al., 2011). Future research should be focused to examine whether ARF-BP1 is sufficient to reverse the MDM2 inhibition of cancer. During stress, USP29 removes the polyubiquitin chain from p53 thus stabilizing it and induces p53 dependent apoptosis (Liu et al., 2011; Figure 6). Little is known about the expression pattern and biological functions of USP42 as a DUB for p53. In the initial phases of a stress response, USP42 deubiquitinates p53 by forming a direct complex with p53. It does not affect the basal levels of p53 in unstressed cells and its depletion delays the activation of p53 target genes (Hock et al., 2011). USP9X deficient cells promoted p53 degradation suggesting it to be considered as a potential therapeutic modulator in p53-related tumors (Liu et al., 2015).

OTU family members also play a role in regulating p53. OTUB1 modulates p53 stability by suppressing the conjugation between MDM2 and its E2 enzyme, UbcH5. Overexpression of OTUB1 induces p53-dependent apoptosis whereas its knockdown attenuates p53 activation (Sun et al., 2012). OTUB1 has recently been known to inhibit MDM2 mediated MDMX ubiquitination leading to enhanced p53 phosphorylation at Ser46 thus promoting mitochondria-mediated apoptosis. In addition, OTUB1 also promotes UV induced apoptosis (Chen et al., 2017; Figure 6). Future study will be focused on knowing about OTUB1 activated by UV damage. Upon DNA damage OTUD5 specifically, deubiquitinates and stabilizes p53 but not the MDM2 (Luo et al., 2013). A recent study showed that in case of stress, programmed cell death 5 (PDCD5) is required to promote the interaction between OTUD5 and p53. OTUD5 thus deubiquitinates PDCD5 at Lys97/Lys98 subsequently regulating p53 levels (Park et al., 2015; Figure 6; Table 8).

**Table 8:** List exhibiting the functions of DUBs involved in p53-mediated apoptosis.

**Name**	**Function**
USP7	Deubiquitination of p53
USP10	Deubiquitination of p53
USP11	Deubiquitination of p53
USP2	Deubiquitination and stabilization of MDM2
USP24	Deubiquitination of p53
USP29	Deubiquitination of p53
USP42	Deubiquitination of p53
USP9X	Deubiquitination and stabilization of p53
OTUB1	Inhibition of MDM 2 mediated MDMX ubiquitination
OTUD1	Deubiquitination of p53
OTUD5	Deubiquitination and stabilization of p53

## UPS as a drug target

The role of ubiquitination and deubiquitination in the development of numerous cancers, immune pathologies, and neurodegenerative diseases and possibilities of targeting them for the development of therapeutics against these diseases have been covered in multiple reviews (Ciechanover, 2006; Hoeller et al., 2006; Reinstein and Ciechanover, 2006; Paul, 2008; Deshaies, 2014; Huang and Dixit, 2016). Mechanistically, the disorders connected with deregulation of UPS can be broadly classified into two groups: (i) that are the eventual outcome of a mutation in a UPS enzyme or a target substrate (loss of function), inhibiting ubiquitination and subsequent degradation of certain proteins, and (ii) that are the outcome of an abnormal or accelerated degradation of the target protein (gain of function; Reinstein and Ciechanover, 2006). The success of the kinase inhibitors has encouraged the pharmaceutical industry to attempt the same strategy in targeting the UPS (Zhang et al., 2009; Knight et al., 2010). However, efforts until now brought about the development of only a handful of inhibitors that have entered into clinical trials, mainly because of the fact that most of the UPS enzymes do not contain a well-defined catalytic pocket, making small molecule difficult to target these proteins. Moreover, multiple protein-protein interactions involved among UPS enzymes are challenging to disrupt with a single inhibitor (Huang and Dixit, 2016). Despite these challenges, there have been studies to find the way to perturb the system to treat diseases. Novel and robust functional *in vitro* assays coupled with structural studies hold great promise for making the system readily accessible for drug discovery and development.

Below we attempt to summarize the involvement of components of the ubiquitin-proteasome system in human diseases and the clinical progress that has been made in targeting the system in hope to developing more therapeutics.

### Targeting the ubiquitin activating/E1 enzymes

In humans, a family of eight mechanistically and structurally-related ubiquitin-activating enzymes for ubiquitin and more than a dozen ubiquitin-like proteins (Ubls) are known (Schulman and Harper, 2009). Human E1 enzymes specific for ubiquitin are referred to as UBA1 and UBA6. An E1 enzyme activates ubiquitin in the presence of ATP for a nucleophilic attack by forming thiol ester bond between C-terminal carboxyl group of ubiquitin and its own cysteine. PYR-41 was the first identified cell-permeable and reversible inhibitor of E1, potentially covalently modifies the active cysteine residue of UBA1 (Yang et al., 2007). However, the specificity of this inhibitor was not assessed in depth against other E1 enzymes.

Another pyrazolidine pharmacophore PYZD-4409, similar to PYR-41, predominantly induces cell death in malignant cell lines, but the precise mechanism of its action remains unknown (Xu G. W. et al., 2010). A small molecule inhibitor of UBA1 which is in active phase I trial is MLN7243^1^ (Millennium Pharmaceuticals Inc.). MLN7243 that had been initially identified with potential antineoplastic activity in advanced malignant solid tumors, has been shown to bind and inhibit UBA1. Inhibition of UBA1, consequently, prevents substrate ubiquitination and degradation. Millennium Pharmaceuticals also reported an adenine sulfamate analog Compound I that blocks E1-dependent ATP-PPi exchange activity and inhibits E1-E2 transthiolation. The covalent ubiquitin-Compound I adduct formed, mimics the ubiquitin-adenylate intermediate and blocks the active site of the enzyme to prevent further recruitment of substrates but its effects on cell viability were not reported. Given that all E1 enzymes activate their cognate ubiquitin or ubiquitin-like proteins mechanistically similarly, adenosine sulfamate analogs may display a novel approach to develop potent and selective E1 inhibitors (Chen et al., 2011).

Like ubiquitination, neddylation, the covalent attachment of NEDD8 (ubiquitin-like protein) to several substrates critically controls cell proliferation and cell death. MLN4924, an inhibitor of the NEDD8 activating (E1) enzyme is already in several phase II clinical trials, showed disruption of neddylation of Cullin, a regulatory scaffold found in multi-subunit Cullin-RING E3 ligases and hence influencing CRL activity leading to the accumulation of both oncoproteins and tumor suppressors such as p27, NRF2, and IκB (Huang and Dixit, 2016). Inhibition of the enzyme by inhibitor MLN4924 induces uncontrolled DNA synthesis during S-phase, promoting DNA damage and cell death in proliferating tumor cells (Soucy et al., 2009; Huang and Dixit, 2016).

### Targeting the ubiquitin conjugating/E2 enzymes

The E2 conjugating enzymes transfer activated ubiquitin onto downstream substrate protein interacting with numerous E3 ligases (Ye and Rape, 2009). Traditionally, E2 conjugating enzymes were to be known as ubiquitin carriers, but recent studies demonstrated that E2 enzymes do not solely dictate the length of the chain and linkage however in several cases also determine specificity toward the incoming substrate (Streich and Lima, 2014; Huang and Dixit, 2016; Stewart et al., 2016). Humans have ~40 E2 enzymes that are involved in the transfer of Ubiquitin or Ubiquitin-like (Ubl) proteins (e.g., SUMO and NEDD8), hence targeting E2s gives more selectivity as compared to E1s and may represent the more viable option as drug targets.

A small molecule, CC0651, identified as an allosteric inhibitor of a human E2 enzyme, Cdc34 binds to the pocket that is present away from the catalytic site, causes a large conformational change in the enzyme structure and interferes with the ubiquitin transfer (Ceccarelli et al., 2011). Despite showing promising results *in vivo*, CC0651 however, fails to make into clinical trials due to difficulties in optimization. Another small molecule inhibitor, NSC697923 blocks the transfer of ubiquitin onto substrates by inhibiting the formation of UBE2N~Ub thioester conjugate in a heterodimer E2 conjugating complex of UBE2N-UBE2V1 (Pulvino et al., 2012). Another compound BAY11-7082 was originally thought to inhibit apoptosis of colon cancer cells by inhibiting TNFα-stimulated phosphorylation of IκBα and thus eventually inhibiting the NF-κB pathway (Pierce et al., 1997). However, a recent study showed that the compound exerts its effect by inactivating the E2-conjugating enzymes UBE2N (Ubc13) and UbcH7 and presumably many more E2 enzymes by covalently modifying their reactive cysteine residues (Strickson et al., 2013; Huang and Dixit, 2016).

### Targeting E3 ligases

E3 ligases family constitutes over 700 members identified or predicted to possess ligase activity (Li et al., 2008). As E3 family of enzymes comprising of the largest number of members that use distinct catalytic mechanisms, inhibiting E3 ligases is expected to possess better specificity with less associated toxicity comparing to targeting E1s, E2s, or proteasomes (Huang and Dixit, 2016). As it is beyond the scope of this review to cover all the E3 ligases involved in various malignancies and disorders and the ongoing efforts to target E3 ligases, we have focused on some of the most promising examples listed below.

#### MDM2

p53 is undisputedly one of the most vital tumor suppressors and a nuclear transcription factor that has been implicated in controlling many essential cellular processes like apoptosis, cell cycle arrest, DNA repair, autophagy, and senescence in response to stress signals. No less than half of the human cancer types express a mutated version of p53 protein (Levine, 1997). Tumors expressing wild-type p53 protein often acquires alternative mechanisms to subdue its activity. One such example is an overexpression of MDM2, an E3 ligase that ubiquitinates p53 (Quesnel et al., 1994; Chène, 2003). Being a negative regulator of p53, MDM2 is emerging as a potential drug target (Huang and Dixit, 2016). A small molecule, RITA (Reactivation of p53 and Induction of Tumor cell Apoptosis) was identified from the chemical compound library that can show toxicity in tumor cell lines in a p53-dependent manner. RITA was shown to abolish the interaction between p53 and MDM2 by binding to p53, thus inhibiting p53 ubiquitination and its proteasomal degradation (Gudkov, 2004; Issaeva et al., 2004). In the same year, Vassilev and colleagues reported Nutlins, a family of cis-imidazoline analogs that occupies the pocket in MDM2 where p53 binds thus inhibiting ubiquitination and degradation of p53 (Vassilev et al., 2004). An orally bioavailable small molecule selective inhibitor, MI-219, binds to MDM2 like in the case of Nutlins and blocks MDM2-p53 interaction, leading to induction of cell cycle arrest in both normal and tumor cells but selective apoptosis only in tumor cells (Shangary et al., 2008). Restoration of wild-type p53 expression by disrupting p53-MDM2 interaction certainly holds great potential in triggering cell death and eliminates tumors *in vivo* but with the p53 mutation occurring frequently in human tumors, it also plays a critical role in tumor evolution by allowing evasion from apoptosis as mutated p53 is no longer ubiquitinated by MDM2 and becomes stabilized (Lukashchuk and Vousden, 2007). The identification of small molecules that reactivates mutant p53 protein opens up the possibilities for the development of more effective drugs against various cancers. Screening a library of low-molecular-weight compounds showing potent activity and selectivity for mutant p53 cell types in anti-proliferation assays identified 3-methylene-2-norbornanone, a compound that restores native conformation to mutant p53 in cells (Reddy et al., 2004). Another independent screening study identified small molecules PRIMA-1 and MIRA-1 that covalently modify the thiol groups of one or more cysteine residues in the mutant p53 core domain, restoring the wild-type conformation, and tumor suppressor function of mutant p53 (Bykov et al., 2005; Lambert et al., 2009).

#### SCF^Skp2^

Another tumor suppressor that found to be altered in many malignancies such as colorectal cancer (Palmqvist et al., 1999), breast cancer (Spirin et al., 1996; Tan et al., 1997) prostate cancer (Cote et al., 1998), lung cancer, and uterine endometrium cancer is p27. p27 is a cell-cycle inhibitor and various mechanistic analyses have shown that the low levels of p27 in various human carcinomas are a consequence of its abnormally enhanced degradation by virtue of Skp2 overexpression (Gstaiger et al., 2001; Li J.-Q et al., 2004). Skp2 functions as a substrate recognition subunit of a complex termed SCF that is formed together with Skp2, Cullin1, Rbx1 (Spirin et al., 1996; Carrano et al., 1999; Gstaiger et al., 2001; Bloom and Pagano, 2003). More mechanistic knowledge into the Skp2 overexpression in various cancers holds great therapeutic potential as inhibiting p27-Skp2 interactions may reverse its malignant phenotype. Various studies have demonstrated that targeting Skp2 in various cancer cells as well as in animal models resulted in the upregulation of p27 level that results into tumor shrinkage and apoptosis (Yokoi et al., 2003; Kudo et al., 2005; Lee and McCormick, 2005). The binding pocket of Skp2, essential for binding of p27 was targeted through virtual screening. Positive hits were confirmed by biochemical and biophysical studies and found to be inhibiting Skp2-p27 interaction and therefore blocking the p27 degradation (Wu et al., 2012).

#### Targeting IAP proteins

c-IAP1/2 and other c-IAP proteins have been linked to numerous human pathologies (such as multiple myelomas) in connection with both canonical and non-canonical NF-κB signaling. Amplification of genes expressing c-IAP1/2 has been associated with a variety of human malignancies, including mammary carcinoma, hepatocellular carcinoma, and medulloblastoma, and in cervical, lung, pancreatic, oral squamous cell, and esophageal carcinomas (Fulda and Vucic, 2012; Popovic et al., 2014). Development of a novel class of small-molecule IAP antagonists that bind to the BIR domains of IAP proteins promotes rapid ubiquitination and proteasomal degradation of c-IAPs and RIP1 inflicting the activation of canonical and noncanonical NF-κB pathways (Varfolomeev et al., 2007; Bertrand et al., 2008). Demonstrating, robust anticancer activity in mouse tumor models, several IAP antagonists (GDC-0152, LCL161, HGS1029, and TL32711) are already in clinical trials and have shown promising results with tolerable toxicity (Fulda and Vucic, 2012; Popovic et al., 2014).

### Targeting the proteasome

Various studies have demonstrated that proteasome inhibition is more toxic in various malignant cell lines as compared to normal cells. *In vivo* studies in cancer cell lines have proven target antagonism of proteasome inhibitors at high doses level with low toxicity to normal cells (Richardson et al., 2006). At present, three FDA approved proteasome inhibitors Bortezomib (PS341, Velcade)- a peptide boronate, Carfilzomib (Kyprolis)- a peptide epoxyketone, and Ixazomib (MLN9708, Ninlaro®)- another peptide boronate are used routinely in clinical settings for the treatment of multiple myeloma or mantle-cell lymphoma in US and other countries.

Bortezomib was the first proteasome inhibitor to enter clinical trials and approved by FDA in 2003, acts through inhibition of the UPS (Kane et al., 2003; Richardson et al., 2006). Its antitumor effect is attributed to its ability to affect several critical pathways. Inhibition of degradation of abnormal proteins and the inactivation of an antiapoptotic transcription factor, NF-κB signaling are among the few (Reinstein and Ciechanover, 2006; Richardson et al., 2006). Aberrant activation of NF-κB has been linked with a variety of solid tumors (breast, prostate, lung, and pancreatic cancers), in a number of hematopoietic cancers (likes of multiple myeloma, chronic myelogenous leukemia, and Hodgkin's and non-Hodgkin's lymphomas) and multiple human immunological diseases (including incontinentia pigmenti, ectodermal dysplasia coupled with immunodeficiency, chronic autoinflammation, and muscular amylopectinosis; Popovic et al., 2014). *In vitro* treatment of bortezomib in B-cell lines and primary chronic lymphocytic leukemia (CLL) cells has shown to induce and stabilize the Bax accumulation, its translocation to mitochondria and oligomerization (Liu et al., 2008). Accumulation and stabilization of proapoptotic Bax protein sensitize B-cells to TRAIL-induced apoptosis. Currently, over 800 diverse clinical trials are running that uses bortezomib alone or together with another drug for the treatment of earlier-stage multiple myeloma, non-Hodgkin lymphomas (which includes mantle-cell lymphoma), and other solid tumors^2^ (Goy et al., 2005; O'connor et al., 2005; Leonard et al., 2006; Chen et al., 2011).

Carfilzomib (PR-171, Kyprolis), is the second proteasome inhibitor approved by the FDA in the year 2012 that forms a stable and irreversible adduct with the proteasome and inhibits the chymotrypsin-like site in the catalytic subunits of the proteasome. Carfilzomib has been shown as a more potent inhibitor than bortezomib, inducing responses in bortezomib-resistant multiple myeloma (Kortuem and Stewart, 2013). Although very effective, dose-limiting toxicities and development of resistance, bortezomib, and carfilzomib have inspired interest in developing novel proteasome inhibitors that have greater efficacy but improved safety profile (Eldridge and O'brien, 2010).

Development of orally bioavailable second-generation proteasome inhibitors MLN9708 (Ixazomib, Ninlaro®) and CEP-18770 showed proteasome-inhibitory action equivalent to bortezomib but with better pharmacokinetic properties. Ixazomib (Ninlaro®) is the first orally administered proteasome inhibitor approved by FDA is currently being used in the US, Europe and Japan in combination with lenalidomide and dexamethasone in patients with relapsed and/or refractory myeloma (Al-Salama et al., 2017; Manasanch and Orlowski, 2017). Treatment with MLN9708 or its biologically active form MLN2238 (Millennium Pharmaceuticals, Inc.) predominantly inhibits growth and triggers apoptosis in multiple myeloma cells that were already impervious to traditional and bortezomib therapies without influencing regular cells (Chauhan et al., 2011). Although CEP-18770 (delanzomib) showed promising results in promoting apoptosis in human multiple myeloma cell lines and patient-derived cells by suppressing NF-κB–mediated mediated signaling pathways and by inhibiting endothelial cell survival, and RANKL-induced osteoclastogenesis *in vitro* (Piva et al., 2008), development of delanzomib for myeloma was discontinued owing to its dose-limiting toxicities (Vogl et al., 2017). Another proteasome inhibitor, PR-957 prevents experimental autoimmune disease rheumatoid arthritis in the mouse model by acting directly on the low-molecular-mass polypeptide-7 (LMP7), which is the chymotrypsin-like subunit of the immunoproteasome (Popovic et al., 2014). Efficacy of other proteasome inhibitors, including the irreversible epoxyketone Oprozomib (Shah et al., 2015), and the intravenous marine-derived β-lactone-γ-lactam Marizomib (NPI-0052; salinosporamide A; Potts et al., 2011) has been evaluated clinically either as single agent or in combination with other drugs (Manasanch and Orlowski, 2017). Marizomib showed broader and irreversible inhibition of all three 20S proteasome proteolytic activities in various *in vitro* models and is efficacious *in vivo* against multiple myeloma, other hematologic malignancies, and solid tumor models, including colon and pancreatic carcinomas (Potts et al., 2011). Mechanistically, first and second generation proteasome inhibitors triggers apoptosis in myeloma cells that is associated with enhanced expression of proapoptotic genes like p53, p21, Noxa, Puma, and E2F, downregulation of antiapoptotic genes, activation of caspases cascade and the inhibition of NF-κB signaling by blocking degradation of the NF-κB inhibitor IκB.

### Targeting DUBs

Many times, it could be of therapeutic potential to upregulate the degradation of UPS targets such as oncoproteins. The simplest way to achieve this is to inhibit the associated DUBs activity. Like E3s, many DUBs are deregulated and implicated in various diseases (Hussain et al., 2009; Sacco et al., 2010). As a consequence, DUBs represent alternative targets in the UPS for certain malignancies and human disorders. Several studies have shown that genetic or chemical interference with USP7/HAUSP function destabilizes MDM2 and increases p53 levels (Li et al., 2002; Cummins et al., 2004; Li M. et al., 2004). Compound P5091 was identified as a USP7 inhibitor that selectively inhibits USP7 activity both *in vivo* and *in vitro*, and induces p53 and p21 expression. P5091 also induces apoptosis in multiple myeloma cells that are previously impervious to traditional therapies and bortezomib treatment (Chauhan et al., 2012). Another study identified compound NSC 632839 (compound F6), which inhibits purified USP2 and USP7 enzymes *in vitro* at IC50 values of 45 and 37 mM, respectively (Nicholson et al., 2008). Another small molecule b-AP15 was shown to inhibit two 19S regulatory-particle–associated deubiquitinases, ubiquitin carboxyl-terminal hydrolase 5 (UCHL5) and ubiquitin specific peptidase 14 (USP14). Treatment with b-AP15 results in accumulation of polyubiquitinated substrates and inhibition of tumor progression in solid tumor models *in vivo* as well as inhibition of organ infiltration in an acute myeloid leukemia model (D'arcy et al., 2011). Another study identified a small molecule, HBX 41,108, a cyano-indenopyrazine derivative that inhibits USP7 activity in a reversible manner both *in vitro* and in cancer cells with an IC50 value in the submicromolar range. Treatment with HBX 41,108 activates the transcription of p53 target genes without inducing genotoxic stress and p53-dependent apoptosis subsequently resulting in inhibiting tumor proliferation (Colland et al., 2009). Another small molecule; compound 1 identified as a selective inhibitor for USP7 and also showing moderate activity against USP47, *in vivo* elevates the p53 level and induces p53-mediated apoptosis in cancer cell lines (Weinstock et al., 2012). Recently, two more compounds, FT671 and FT827, were shown to inhibit USP7 both *in vitro* and *in vivo* with high affinity and specificity with IC50 values in the range of nanomolar. Crystal structures of the inhibitors in complex with an inactive USP7 apo-enzyme revealed that both compounds target a dynamic pocket near the catalytic center of the enzyme, which is not present in either of the apo- or ubiquitin bound structures of USP7 and also differs from other DUBs. Similar to the knockdown studies of USP7 in colorectal carcinoma (HCT116) or bone osteosarcoma (U2OS) cell lines, compound FT671 elevates the p53 level and induces transcription of p53 target genes including Puma, p21, and MDM2. Overexpression of p53 correlated with the increase in MDM2 ubiquitination and its degradation, which is initially counterbalanced by initial induction of p53-induced MDM2 expression (Turnbull et al., 2017).

## Discussion and future perspectives

The UPS controls a huge number of proteins and nearly every biological process in almost every cellular compartment. Through covering apoptotic pathways, we have summarized how enzymes of the UPS control the cell death. Given that thousands of proteins of the cellular proteome are ubiquitinated at some point in their lives, numerous pathologies and malignancies can be linked directly or indirectly to deregulation of the system. Recent experimental evidence strongly suggests that pharmacologic manipulation of the UPS using specific inhibitors would possibly influence the end result in certain pathologies likes in cancer, asthma, brain infarct, and autoimmune encephalomyelitis. The first drug to be used in clinical trials based on UPS inhibition was bortezomib, a proteasome inhibitor that obstructs the complete ubiquitin system. The success of bortezomib has prompted pharmaceutical companies to invest in the development of other UPS-directed drugs as potential mechanism-based therapies that have greater efficacy but fewer side effects. However, despite a large amount of literature available on the mechanisms of action of the system and its pathophysiologic characteristics, the unknown far exceeds what is presently known. Thus, the UPS stays to be a comparatively unexplored world for therapeutics to its potential, but one that has the promise of significant advancements in the near future. What we hope to achieve in the near future is the better understanding of the mechanisms and identification of the substrates involved and their specific E2, E3, and DUBs that holds promise for the development of highly specific mechanism-based, more efficient and less toxic drugs for various diseases. As briefly discussed in this and in various other reviews (Petroski and Deshaies, 2005; Hoeller et al., 2006; Reinstein and Ciechanover, 2006; Hoeller and Dikic, 2009; Vucic et al., 2011; Fulda and Vucic, 2012; Deshaies, 2014; Huang and Dixit, 2016), various novel strategies are in progress for the development of drugs targeting the ubiquitin-proteasome system.

## Author contributions

IG, KS, and NV: Performed a bibliographic search; NV: Designed all the figures; IG and KS: Made all the tables; IG, KS, NV, and SK: Wrote the manuscript; All authors read and approved the manuscript.

### Conflict of interest statement

The authors declare that the research was conducted in the absence of any commercial or financial relationships that could be construed as a potential conflict of interest.

## Footnotes

^1^ClinicalTrials.gov, search term,“MLN7243” [https://clinicaltrials.gov/ct2/results?cond=&term=MLN7243&cntry1=&state1=&recrs=].

^2^ClinicalTrials.gov, searchterm “bortezomib”. [https://clinicaltrials.gov/ct2/results?cond=&term=bortezomib&cntry1=&state1=&recrs=].
